# Performance of Oral Cavity Sensors: A Systematic Review

**DOI:** 10.3390/s23020588

**Published:** 2023-01-04

**Authors:** Leonardo de Almeida e Bueno, Man Ting Kwong, Jeroen H. M. Bergmann

**Affiliations:** 1 Department of Engineering Science, University of Oxford, Oxford OX1 3PJ, UK; 2 Guy’s and St. Thomas’ NHS Foundation Trust, St. Thomas’ Hospital, Westminster Bridge Rd., London SE1 7EH, UK

**Keywords:** intraoral, measurement, biosensors, biomedical monitoring, mouth

## Abstract

Technological advancements are enabling new applications within biomedical engineering. As a connection point between the outer environment and the human system, the oral cavity offers unique opportunities for sensing technologies. This paper systematically reviews the performance of measurement systems tested in the human oral cavity. Performance was defined by metrics related to accuracy and agreement estimation. A comprehensive search identifying human studies that reported on the accuracy or agreement of intraoral sensors found 85 research papers. Most of the literature (62%) was in dentistry, followed by neurology (21%), and physical medicine and rehabilitation (12%). The remaining papers were on internal medicine, obstetrics, and aerospace medicine. Most of the studies applied force or pressure sensors (32%), while optical and image sensors were applied most widely across fields. The main challenges for future adoption include the lack of large human trials, the maturity of emerging technologies (e.g., biochemical sensors), and the absence of standardization of evaluation in specific fields. New research should aim to employ robust performance metrics to evaluate their systems and incorporate real-world evidence as part of the evaluation process. Oral cavity sensors offer the potential for applications in healthcare and wellbeing, but for many technologies, more research is needed.

## 1. Introduction

The development of sensor technologies fuelled the evolution of biomedical sciences [1,2]. Ubiquitous devices allowed continuous and real-time monitoring of individuals in the most diverse settings and applications [3]. As a connecting point between the outer environment and human systems, the oral cavity offers unique opportunities for non-invasive monitoring not found in other conveniently available locations in the body.

The oral cavity is an important part of the human body. It is an accessible invasive anatomical site with a direct linkage to the digestive and respiratory systems [4,5]. It also has proximity to the brain and offers a connection to the circulatory and nervous systems [6]. The oral cavity is a gateway for air, food, medications, infectious microbes, and toxic substances to the body [7]. Besides being the obvious site for maintaining dental health, the oral cavity also has systemic importance for human health. Several systematic chronic diseases (such as rheumatoid arthritis, atherosclerosis, and Alzheimer’s disease) have been connected to oral diseases [8,9].

The oral cavity has been the focus of dentistry for decades. However, several medical fields are now looking to harness the prognostic potential of this site by placing sensors in it. Saliva and breathed air are rich biologic media that can be analyzed for potential diseases, infectious agents, or metabolic changes [10,11,12]. Respiration can also be leveraged for non-invasive disease diagnosis through the detection of certain biomarkers [11,12,13]. The aforementioned proximity to the brain and nervous system and the direct role in speech, swallowing and breathing makes the oral cavity a further promising site for monitoring and diagnosing neurological pathologies.

Nonetheless, the oral cavity is a harsh environment for sensors. The constant contact with air, matter, metabolites, and enzymes greatly limits the sensor technologies, materials and approaches that can be applied inside the mouth. The presence of bones, teeth and hard and soft tissues provides challenges in terms of sensor placement and design. The wet environment of the oral cavity also demands the use of wet materials or wet-endurable materials [14,15,16]. Integration of these materials with different solid substrates is a requirement for the stable performance of some intraoral sensors [17]. Furthermore, considerations in terms of hygiene are paramount for gaining regulatory approval. 

Advances in sensor technologies have progressed the research and development of oral cavity sensors [18,19]. Whilst early stage testing and validation of body sensors can be performed on models (including computational models, animal models and biomimetic models), a successful sensor will need to be tested in humans. The testing will need to fit the requirements, which can differ depending on the setting (e.g., clinical, sports, or home settings).

Clinical trials are crucial to measuring the safety and effectiveness of devices, confirming, validating, and complementing data from the bench and/or animal testing [20]. Human trials are also important for identifying and mitigating usability errors due to human factors, a mandatory evaluation of medical devices [21]. Contrary to the belief that human factors are easy or “common sense,” the numerous product recalls and adverse events reinforce the need to test sensor systems for a wide range of users to account for person-to-person variation [22]. Most studies explore a research concept and only reported laboratory results, as the oral cavity is an extremely challenging location for real-world (clinical) testing. Furthermore, clear reporting on performance metrics is required to allow for comparisons between developed oral cavity sensor techniques.

This review systematically identifies and assesses which sensor technologies were successfully employed in the oral cavity and how they performed. This work will help identify challenges and possible research gaps associated with the development of oral sensors. The main research questions are:

1. What sensor technologies have been tested in the human mouth?
2. Which fields of study develop sensor technologies for the mouth?
3. What is the performance of these sensors in terms of accuracy and/or agreement?

## 2. Methods

This systematic review was structured following the Preferred Reporting Items for Systematic Reviews and Meta-Analyses (PRISMA) guideline [23,24]. The review was registered with PROSPERO (CRD42020199089).

The literature search was conducted in Medline, Embase, IEEEXplore, Scopus and Web of Science. The search strategy included the keywords: (“Monitoring device” OR “Transducer” OR “Sensor” OR “Microsensor” OR “Sensing device” OR “Biosensor” OR “Biosensing” OR “Instrumented”) AND (“Oral” OR “Mouth” OR “Intraoral” OR “Mouthguard” OR “Tooth”). The search terms were selected and combined to obtain all papers related to measurement systems and sensor placements inside the oral cavity of humans. The final search included all English-language peer-reviewed papers published until the 12th of October 2022.

All peer-reviewed journal articles evaluating the performance of sensors placed in the oral cavity were included if they consisted of trials conducted on living humans. Studies based on animal testing were excluded. The performance evaluation needed to be the primary or secondary outcome of the study for inclusion. Extended abstracts, conference papers, posters and unpublished studies were not accepted for further review. No restrictions based on clinical settings or participant groups were imposed. Only methods where the actual sensing instrument is placed inside of the oral cavity were selected, even if part of the signal processing was performed outside of the mouth. Studies in which the sensing device is positioned outside of the mouth, but connected to the oral cavity through pipes, tubes, or any other channels, were excluded. Studies must contain an outcome measure–methodology alone does not suffice, and they must report estimates of accuracy/agreement, including measures of statistical uncertainty using valid statistical methods. The list of appropriate statistical methods was built following [25,26,27]. Accepted methods for estimation of agreement were Bland–Altman plot/limits of agreement analysis, intraclass correlation coefficient, Lin’s concordance correlation coefficient, British standards reproducibility/repeatability coefficient, Kappa and Weighted kappa coefficients. Accepted methods for estimation of accuracy were accuracy, sensitivity, specificity, false-positive rate, false-negative rate, mean square error/deviation, root mean square error/deviation, mean absolute error/deviation, and metrics derived from those.

All eligible studies that could not be accessed through library services were attempted to be obtained by contacting the corresponding author.

The primary outcome was to describe the current availability and performance of oral cavity sensors tested in living humans. The secondary outcomes were to assess the quality of the methodology reporting concerning the adapted Specialist Unit for Review Evidence (SURE) [28] and the Quality Assessment of Diagnostic Accuracy Studies–2 (QUADAS-2) [29]. A total of 13 items were selected for the quality assessment. 

The titles, abstracts and full texts were screened by two reviewers (LAB and MTK). Clearly unrelated studies were rejected. Quality assessment was performed by LAB and confirmed by a second reader (MTK). At any stage, in cases of discrepancies unresolved by discussion, the third reviewer (JHMB) was consulted.

Due to the heterogeneous nature of study designs, results and outcomes, a formal quantitative analysis would not be appropriate. Instead, similar numerical reports were combined to provide a narrative summary.

## 3. Results

**Figure 1:**
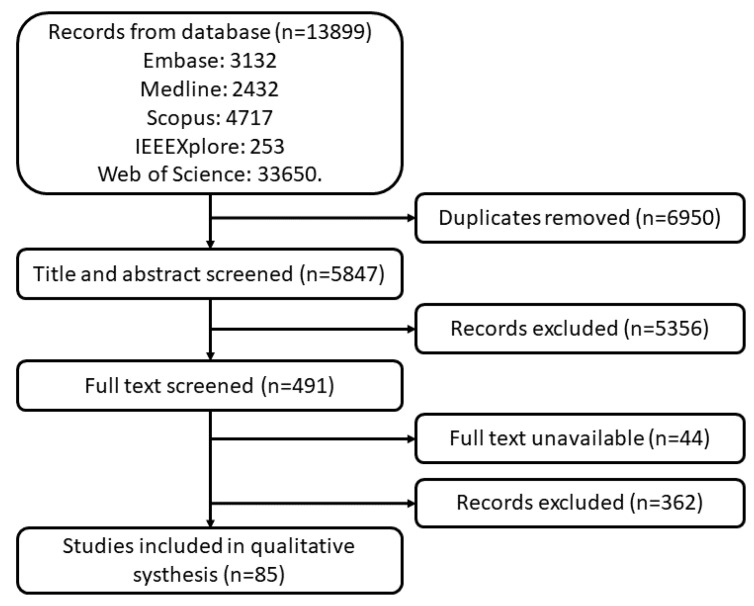
PRISMA flow diagram of search results.

**Figure 2:**
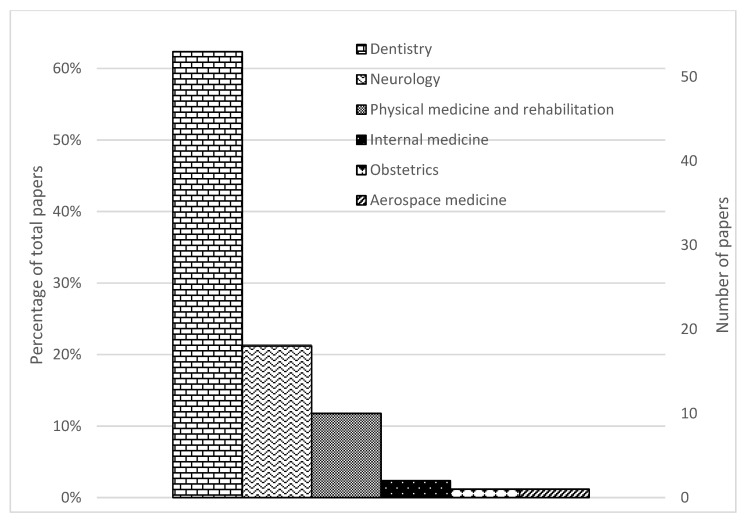
PRISMA flow diagram of search results.

**Figure 3:**
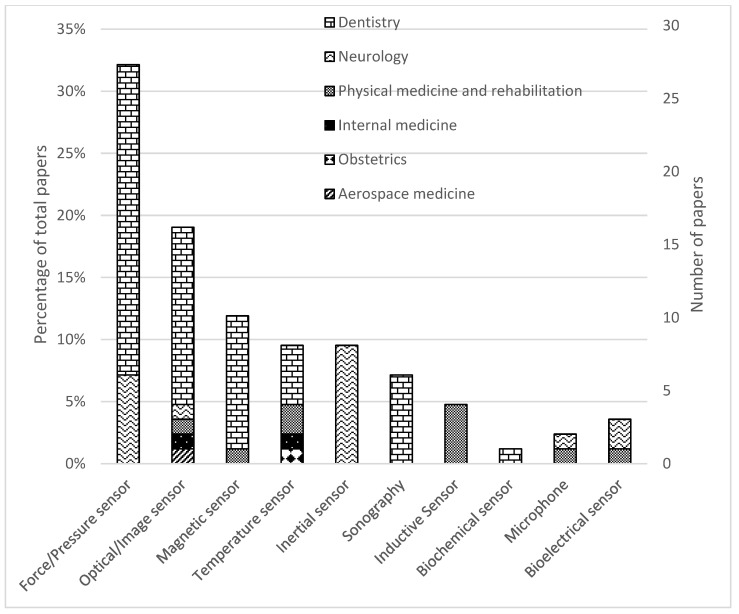
Distribution of papers by field of study and sensor technology.

A total of 13,899 studies were found in the searched databases. After duplicate removal 6950 papers went through the title and abstract screen and 5356 papers were excluded. The remaining 491 papers were fully read, and 85 papers were finally selected. The PRISMA flow diagram of search results is shown in Figure 1. Figure 2 and Figure 3 provide the breakdown of the fields of study and sensor technologies that were addressed by the identified literature.

Forty-four full texts were not retrieved. Attempts to contact the authors were made, but no responses were received. 

### 3.1. Quality Assessment

Several aspects regarding the paper quality were assessed in this review (see Table 1). Across all literature, 47 papers (47/85, 55%) reported an appropriate reference standard for the human tests. The main reason for this is that some measurements were validated in vitro using calibration machines. In total, 26 (26/85, 31%) of the papers did not report ethical approval, but it should be noted that 10 papers were published before the 5th revision of the Declaration of Helsinki [30] in the year 2000. It was also found that 22 papers (22/85, 26%) did not adequately report the limitations of their research.

### 3.2. Fields of Study

The identified studies were classified according to the definitions by the American Medical Association [31] and the American Dental Association [32]. Around 62% (53/85) of the studies were in dentistry, 21% (18/85) were in neurology, 12% (10/85) were on physical medicine and rehabilitation, 2% (2/85) was on internal medicine, 1% (1/85) was on obstetrics and 1% (1/85) was on aerospace medicine.

### 3.3. Sensor Technologies

Categorizing the papers based on sensor technology we observe that 27 studies (27/85, 32%) employed force or pressure sensors (21/85 in dentistry and 6/85 in neurology). Sixteen studies (16/85, 19%) employed optical or image sensors, including x-ray (12/85 in dentistry, 1/85 in neurology, 1/85 in physical medicine and rehabilitation, 1/85 in aerospace medicine). A total of ten studies (10/85, 12%) employed magnetic sensors (9/85 in dentistry, 1/85 in physical medicine and rehabilitation). Eight studies (8/85, 9%) employed inertial measurement units for neurology. Eight studies (8/85, 9%) used temperature sensors (4/85 in dentistry, 2/85 in physical medicine and rehabilitation, 1/85 in internal medicine, and 1/85 in obstetrics). Seven studies (6/85, 7%) used sonography in dentistry. Four studies (4/85, 5%) used inductive sensors for physical medicine and rehabilitation. Three studies (3/85, 4%) tested bioelectrical sensors (2/85 in neurology, 1/85 in physical medicine and rehabilitation). Two studies (2/85, 2%) tested microphones (1/85 in neurology, 1/85 in physical medicine and rehabilitation). One study (1/85, 1%) tested a biosensor for dentistry. A breakdown of (i) sensor technologies, (ii) their applications, (iii) the metrics reported and (iv) the considerations me in thereviewed papers can be found in Table 2.

### 3.4. Study Settings

About half of the studies (41/85, 48%) had a small sample size with 20 or fewer subjects. On some occasions this did not reflect on the size of the dataset acquired, as multiple measurements were taken from individuals under different conditions or separate sessions.

A total of 42 studies (42/85, 49%) were performed in academic settings with 31 studies including only healthy adults, five studies (5/85, 6%) included participants with some health or dental condition, four studies had mixed populations (4/85, 5%), and one study was conducted on a healthy paediatric sample population (1/85, 1%). Thirty-five studies (35/85, 41%) were performed in a clinical setting including university hospitals, with 32 studies (32/85, 38%) being performed with subjects who suffered from a health or dental condition and two studies performed with only healthy volunteers (2/85, 2%). Eight studies (8/85, 9%) were performed in a sport setting with a group of healthy volunteers. One study (1/85, 1%) did not specify the study population.

### 3.5. Agreement and Accuracy

A total of 31 studies (31/85, 36%) mentioned agreement metrics. Seven studies (7/85, 8%) reported Bland–Altman analysis, 16 studies (16/85, 19%) reported intraclass correlation coefficients, 8 studies (8/85, 9%) reported Kappa analysis and one study (1/85, 1%) applied concordance correlation coefficient.

In total, 62 studies (62/85, 73%) reported accuracy metrics. Twenty-five (25/85, 29%) studies reported classification accuracy in terms of predictive accuracy and derived metrics. Thirty-seven (37/85, 44%) studies reported accuracy in terms of error rates.

#### 3.5.1. Dentistry

A total of 53 studies were found in the area of dentistry and an overview of paper characteristics is given in Table S1 of the Supplementary Material.

Four studies (4/85, 5%) within endodontics reported the accuracy of modified pulse oximeters in diagnosing dental pulp vitality. Reported accuracies were between 98.75% and 100% while sensitivities ranged between 97.5 to 100% and specificity was found to always be 100% [51,52,53,54].

One study (1/85, 1%) used blood perfusion images to diagnose subsurface cancer. The imager had an overall sensitivity of 96.6% and specificity of 100%. Accuracy in differentiating specific cancer cells from normal tissue varied between 96.6 and 100% [65].

One study (1/85, 1%) used a biochemical sensor to measure salivary pH. The study identified, embedded a miniature pH sensor in a denture and tested it on a 69-year-old woman for 7.5 h. The system presented a calibration error of a maximum of 0.15 pH between pH 5 to 9 (maximum 3%), and up to 0.42 pH for pH ranging from 2 to 12 (maximum 21%) [107].

Five studies in prosthodontics (5/85, 6%) used magnetic systems to perform resonance frequency analysis, a technique where a peg screwed or glued to a tooth (or implant pin) is vibrated using controlled pulses until a mechanical resonance frequency is found [108]. Three studies published by the same group reported agreement metrics for the Osstell devices for measuring implant stability through resonance frequency analysis. Using the intraclass correlation coefficient the studies reported repeatability and reproducibility for the transducer between 0.96 and 0.98 [68,69,70]. Two studies reported sensitivity and specificity of the use of resonance frequency analysis in the diagnosis of tooth ankylosis (specificity of 100% and a sensitivity of 20 to 53.3% depending on measurement direction) [71] and implant failure (sensitivity of 95.2% and specificity of 71.4% with the optimal threshold value) [67].

Seven studies in oral radiology (7/85, 8%) describe imaging technology in dentistry for assessing dental health and diagnosing dental caries and bone defects. Two studies reported the diagnostic accuracy of an image sensor using near-infrared light transillumination for proximal caries detection in permanent teeth. One reported sensitivity of 99.1%, specificity of 94.1%, and accuracy of 97.1% [57]. The study reported Cohen’s kappa coefficient of 93.9% for assessing agreement between caries detection readings [57]. The other study reported sensitivities of up to 84.8%, specificities of up to 97.1%, and accuracies of up to 96.9% for detecting different caries when comparing near-infrared radiology to bitewing radiology. This study also reported sensitivities of up to 88.8%, specificities of up to 97%, and accuracies of up to 99.4% for detecting different caries when compared to clinical direct observation. Agreement between compared methods yielded Kappa coefficients between 0.24 and 0.86 [63]. Three studies used intraclass correlation coefficients to measure the reliability of digital intraoral radiography for a dental assessment. A photostimulable phosphor storage plate x-ray sensor had up to 98.1% agreement with clinical practice in detecting bone defects [60], and up to 51% of observers agreed with the potential of vertical bitewing radiographs in detecting caries when compared with horizontal bitewings [61], while specialists’ agreement on overall image quality superiority from a complementary metal-oxide-semiconductor (CMOS) sensor over a charge-coupled device (CCD) was 66% (95% CI 30% to 87%) [56]. Vertical bitewing images had agreement varying from 23.4 to 51% when compared to horizontal images and clinical observation in identifying caries and bone loss. One study had two specialists identifying artefacts in intraoral periapical and bitewing radiographs. Observers’ agreement resulted in a Kappa coefficient of 99%, operator errors were 70.4%, while plate errors were 19.3% and scanning errors 10.3% [59]. One study reported an agreement of 53.7% with Cronbach Alpha of 84.2% between two specialists for the measurement of external root resorption in the paediatric population. The mean error was 0.73 ± 2.16 mm while the mean absolute error was 1.81 ± 1.40 mm [58].

Four studies (4/85, 5%) applied magnetic sensors in orthodontics. Three studies (3/85, 4%) published by the same group investigated the use of four to eight magnetic sensors embedded in epoxy resin for measuring tooth displacement in orthodontic treatments. The studies reported the calibration/linearity error of the system in measuring the displacement of a tooth. A digital micro-gauge sensor placed in a motorized testing machine was used for calibration. The studies reported errors ranging from 0.2% to 10% depending on the number of sensors and data processing used [72,73,74]. The errors derived from temperature variation were also explored and it was found that displacement measurements varied between 0.20 and 0.33 µm/°C [73]. One study (1/85, 1%) used two tri-axial magnetic sensors placed in the oral-cavity, a controlled magnetic field and a soft polyurethane rubber sample to measure chewing force. The root mean squared error of 1.39 N between the compression pressure tested on the rubber sample and the chewing force estimated from the magnetic sensors [109].

Four studies (4/85, 5%) applied temperature sensors in dentistry. Three studies identified (3/85, 4%), applied thermosensitive microsensors to record the wear time of a sleep apnoea oral appliance. The sensors are encapsulated in medical resin or silicone and are designed to record the temperature at fixed intervals (in minutes) over a period of several months. The temperature records are used to identify how long users wore the appliance in the mouth. All studies reported the under-recording error as their accuracy metric. Recorded wear time had an error varying from 1.70 to 4.2% depending on the device used, as well as the monitoring period [77,79,80]. One study used Bland–Altman analysis and intraclass correlation coefficients as reliability metrics when comparing the digitally registered used time with patient-reported use times. The Bland–Altman limit for digitally registered usage was −0.17 (95% CI: 1.47 to −1.81) hours and the intraclass correlation coefficient was 0.847 (95% CI: 0.834–0.859) for digital and manual record agreements [80]. As a secondary outcome, one study also found that palatal sensors registered a wider temperature range than sensors in the lower buccal sulcus [79]. One study (1/85, 1%) used a thermocouple to measure the temperature difference between intact and gold-restored teeth during intake of hot and cold drinks. The study reported an error of 0.4% when calibrating the sensor between 10–40 °C in a controlled water bath [83].

Thirteen studies (13/85, 15%) were interested in quantifying bite strength in adult patients and healthy adult and paediatric populations. Seven studies reported calibration error and those four studies assessed accuracy drifts due to temperature [35,43], dynamic forces [110], and force direction [110]. Calibration errors reported were less than 1% [36,41,111], around 2% calibration [38,43], and above 4% [33,34]. The error values are not directly comparable because of the variable sensor designs, testing conditions, and error calculations used. Five studies used intraclass correlation coefficient to measure the repeatability of bite force measurements using force sensing resistors (ICC = 0.93) [46], a miniature load cell (ICC = 0.719) [112], or bite forks (ICC = 0.3–0.64 for inter-observer reliability and ICC = 0.63–0.96 for intra-observer reliability) [47,48,109]. One study used Kappa analysis to calculate inter-device reliability (Kappa = 0.8132 ± 0.0544 and 0.8303 ± 0.0538) [109]. One study employed Bland-Analysis to calculate the reliability of a bite force sensor. The study reported 99.5% reliability but did not present any Bland-Analysis plots [113].

One study (1/85, 1%) used a capacitive-type pressure-mapping sensor to classify oral hypofunction in the elderly population. The authors identified low occlusal force with a sensitivity of 75 to 79% and specificity of 75 to 81% [50].

Four studies (4/85, 5%) were interested in measuring soft-tissue forces on teeth. One study employed an intraclass correlation coefficient to quantify the reliability of a beam-type pressure sensor and a diaphragm-type pressure sensor for measuring lip pressure on teeth. The reported ICC ranged from 0.86 to 0.98 for the beam-type sensor and 0.97 to 0.99 for the diaphragm-type sensor [37]. The other three studies reported calibration errors ranging from 1.5% to 4% [35,114,115].

Two studies (2/85, 2%) were interested in diagnosing sleep bruxism based on mouth-clenching patterns. One study reported diagnostic accuracy based on data collected from one healthy subject (sensitivity ranging from 80 to 100% and specificity from 75 to 100%) [49]. Another study used a customized retainer with a pressure sensor in eight subjects to classify bruxism events and reported accuracy of 82.2%, f1-score of 72.4%, sensitivity of 66.5%, specificity of 90.7%, positive predictability of 84.4%, and negative predictability of 83.8% [39].

Another paper (1/85, 1%) quantified the force exerted by a laryngoscope, on the soft tissue of the mouth, during transoral surgery [40]. The study employed force-sensing resistors embedded in a custom-designed laryngoscope cover. The system was calibrated in a specially designed machine and calibration error was accessed in both static and dynamic conditions. The study also evaluated errors due to prolonged application or off-centred forces. A calibration error of less than 5% was mentioned [40].

A total of six papers (6/85, 7%) investigated the performance of sonography in humans. Four studies evaluated the use of ultrasound systems for assessing soft tissue in the mouth, while two studies evaluated the use of ultrasound for assessing oral cancer.

Three studies (3/85, 4%) reported agreement metrics on bone loss and soft-tissue identification. One study used the intraclass correlation coefficient to assess the reproducibility of measurement of buccal bone loss around implants using a linear 12.5 MHz ultrasound [95]. Measurements of moderate bone loss levels had an intraclass correlation coefficient of 0.76 to 0.81 while assessment of marginal bone loss at normal and advanced bone loss levels had an intraclass correlation coefficient of 0.63 to 0.73. The method error ranged from 4.2 to 6.6% [95]. One study evaluated the performance of a new hockey stick transducer in measuring periodontal pockets. Bland–Altman analysis demonstrated that 95% of the replicates fell within 3.2 mm of the differences between the new transducer and the current clinical reference. The agreement between rates was 0.61 to 0.90 [92]. Another study designed a 25 MHz brightness-mode ultrasound transducer and applied Cohen’s kappa coefficient to evaluate its reliability in distinguishing soft tissues in the mouth [94]. The inter-observer agreement to obtain a correct positioning of the probe for interpretable images was κ = 0.90 (*p* < 0.05), to distinguish the bone level κ = 0.97 (*p* < 0.05), to distinguish the marginal gingiva was κ = 0.90 (*p* < 0.05), to visualize the mucogingival line was κ = 0.79 (*p* < 0.05) [94].

Three studies (3/85, 4%) reported predictive accuracy as accuracy metrics for detecting peritonsillar abscess (100%, 95% CI = 75 to 100%) [97], for diagnostic of oral (100%, specificity 91%, and predictive value 94%) and oropharyngeal cancer invasion in the mandibula (sensitivity 77%, specificity 85%, and predictive value 81%), and to guide the need for neck dissection for cancer treatment (positive predictive value of 36.7%, identifying 63.3% of neck dissections as negative) [93]. One paper (1/85, 1%) reported the mean measurement error of tongue cancer tumour thickness, with measurements made using a 15–7 MHz linear ultrasound. These were then compared against histological findings. The average ultrasound measurement error established was 1.28 mm [96]. This study also generated Bland–Altman plots, but precise results could not be extracted from the figures and the plots were not discussed in the text.

#### 3.5.2. Neurology

A total of 18 studies were found for neurology. The study characteristics of these papers are given in Table S2 of the Supplementary Material.

One of the studies found (1/85, 1%) used an intraoral microphone to track breathing during sleep and diagnose obstructive sleep apnoea. It compared the automatic classification of apnoea (and snoring events) based on audio data captured by a tracheal microphone with that of an oral microphone [105]. The oral microphone captured sound frequencies on the spectrum around 1 kHz, which were not present in the data of the tracheal microphone. The oral sensor had a sensitivity of 96.6%, a positive predictive value of 96.6% and a false negative rate of 3.4%, while the tracheal microphone had a sensitivity of 72.9%, a positive predictive value of 97.7% and a false negative rate of 27.1% [105].

Research (1/85, 1%) was also conducted to explore measurements of blood oxygen levels in the mouth for application in sleep apnoea [55]. They found the accuracy of a mouth photoplethysmogram when compared to a fingertip pulse oximeter in awake healthy volunteers, to be 99% [55].

Six studies (6/85, 7%) focused on using pressure sensors for measuring tongue-palatal forces related to swallowing and speech. One paper reported on the classification accuracy (from 93% to 96%) of swallow and non-swallow events, based on the pressure of the tongue on the palate [116]. A second paper showed that a similar sensing technique could yield a sensitivity of 60–87% and a specificity of 71–94% for diagnosing dysphagia in stroke patients [117]. A third study compared sensors for measuring oro-lingual pressures during speech (maximum errors of 8–16% depending on the sensor) [42]. Finally, three studies used the intraclass correlation coefficient to measure the repeatability of tongue-palatal pressure measurements (ICC ranged from 0.687 to 0.956 depending on the conditions) [44,45,118]. One study also reported a standard error of measurement of 14.2% and a minimal detectable change of 38.5% [118].

Two studies used bioelectrical sensors in swallowing and speech. One study used Cohen’s kappa and reported an agreement of 95.6% to 95.8% between trans membranous EMG and needle EMG for assessment of oral cavity muscles (Prevalence-adjusted-bias-adjusted kappa of 0.91 to 0.92) [103]. Another study assessed neuromuscular pathology in the oral cavity and the conduction properties of the tongue (0.1% accuracy) [102].

A total of eight papers (8/85, 9%) investigated the application of microelectromechanical system (MEMS) inertial sensors in the mouth with the intent to monitor head impacts.

One study used the concordance correlation coefficient to evaluate the reliability of commercial mouthguards in impact tests with anthropomorphic test devices (ATDs). Mouthguards were found to have an overall concordance correlation coefficient ranging from 0.80 to 0.97 [86].

Four studies reported normalized root mean square errors for linear and angular accelerations and velocity. Three studies reported errors from impacting anthropomorphic test devices (outcomes ranged from 4.08 ± 1.56% to 9.9 ± 4.4% for linear acceleration, from 9.08 ± 2.98% to 9.7 ± 7.0% for angular acceleration, and 4.39 ± 1.20% to 10.4 ± 9.9% for angular velocity) [88,90,119]. One study reported errors from video-tracked kinematics (15.5 ± 6.0% for anterior-posterior linear acceleration, 18.1 ± 10.0% for inferior–superior linear acceleration, and 12.2 ± 7.0% for sagittal angular velocity), and also reported relative displacement error of the mouthguard in comparison with an earplug reference point (errors of displacement within 1 mm. More than 90% of the errors were within 0.5 mm) [87].

Five studies (5/85, 6%) reported accuracy in detecting video-validated head impacts as accuracy metrics. Mouthguards were found to have an overall sensitivity ranging from 69.2% to 100% and a positive predictive value ranging from 55.0 to 96.4% depending on the device, minimum impact threshold, and data processing technique [85,86,88,89]. Using a series of untuned classifiers, one study reported an average true positives rate of 77.84% and a true negative rate of 89.55% [91]. The best classifier identified was an XGBoost-based model with true positive rates ranging from 94.67 to 100% and true negative rates ranging from 95.65 to 96.83% depending on the used dataset [91].

##### Physical and Rehabilitation Medicine

Six studies (6/85, 7%) evaluated the accuracy of tongue-computer interfaces for severely paralyzed individuals (see Table S3 of the Supplementary Material). Four papers used inductive sensor keys fixed on a palatal plate activated by a ferromagnetic activation unit either glued or pierced to the tip of the tongue. One paper used computer vision and an endoscope camera attached to a mouthguard to identify tongue movements [66]. One study applied four 3-axis magnetic sensors to track the movements of a magnetic tracer glued on the tongue [75].

Three of the six papers used a throughput metric based on movement speed, and accuracy to evaluate generic typing performance. This metric was used to identify the best system layout for typing and tracing tasks. The other two studies used error rates to quantify the performance of the systems in error-free writing and moving cursors and select targets on a screen. For the inductive system, the overall throughput in typing with the keyboard keys was 1.73 bits/s [98], for typing with the mousepad it was 0.60 bits/s [99]. The magnetic system had an overall throughput of 1.25 bits/s in virtual maze navigation and tapping tasks [75].

Two studies out of the six studies reported average correct characters type per minute e and the overall error rate as metrics of performance in typing tasks. The endoscope interface had an average error-free typing rate of 8.152 correct characters/min (maximum 19.34 correct characters/min) and an overall error of 4.95% [66] while the inductive system average performance of 11.6 correct characters/min, with mean error rates ranging from 0 to 36.2% in error-free text writing [101]. One of them reported root mean squared error as low as 0.97 mm (6.5%) when used as a joystick for moving a cursor in the computer [100]. One also tested for operational errors and involuntary activations of the system due to speech and temperature errors [99].

One study (1/85, 1%) assessed the mean error rate and the accuracy of a palatal electrotactile display for rehabilitating tongue motor control individuals with neurotraumas and neurological disorders. The participants identified the stimulation signals with 92.5% accuracy. The mean error reduced from 3.97 ± 0.11 to 0.53 ± 0.19 with proper training [104].

Bland–Altman’s plots were used in one paper (1/85, 1%) to evaluate commercial oral thermometers in estimating body temperature. Oral temperatures were found to have a mean difference to core temperature smaller than −0.1 °C when at rest, 0.9, 0.6 and 0.5 °C colder in recovery, exercise in the heat with wind, and exercise in heat without wind, respectively [81]. Mean differences above 0.5 °C were considered unacceptable by the study. Another study compared the accuracy of a mouthguard instrumented with a digital thermometer when tested in a range of temperatures in a controlled water bath and when worn by a human subject. The study observed that even when the mean absolute error from the experiments is similar (around 0.2 °C) the time to steady state varied significantly (690 s for the water bath compared to 1110 s for the human test) [78].

One study (1/85, 1%) used a microphone embedded in a mouthguard to estimate the perceived exertion of athletes in running exercises. The study reported a normalised root-mean-squared error of 16.20% [106].

##### Internal Medicine

Two studies were found for internal medicine (2/85, 2%). One study evaluated the performance of resonance Raman spectroscopy in measuring tissue haemoglobin oxygenation noninvasively in critically ill patients against central venous haemoglobin oxygen saturation. The study reported a Clarke Error Grid where 84.8% of the data was within the accurate and acceptable grids. The clinical utility yielded an agreement of 0.45 [64]. The other study evaluated the agreement between commercial electronic thermometers against a gold-standard arterial thermometer in estimating the body temperature of patients in critical care (see Table S4 of the Supplementary Material). Oral temperature measured using a battery-free mercury electronic thermometer was found to have a mean error of −0.62 ± 0.34 °C from the arterial temperature reference [84]. The same study reported a mean error of –0.46 ± 0.16 °C of measurement from the axilla compared to the reference using the same device [84].

##### Obstetrics

Table S5 of the Supplementary Material includes the characteristics of one paper (1/85, 1%) that observed a significant difference between oral temperature measurements from electronic and disposable chemical devices in postpartum mothers (mean difference −0.169 ± 0.397 °C) [82].

##### Aerospace Medicine

One study (1/85, 1%) (see Table S6 of the Supplementary Material) applied a near-infrared spectra sensor for measuring the blood oxygen level in pilots as a marker for the detection of hypoxia. The study reported the root mean squared error (RMSE) for measuring blood oxygen level (RMSE = 0.266) and blood flow (RMSE = 0.003) in altitude [62].

## 4. Discussion

The goal of this review was to provide a comprehensive overview of human studies that reported on the performance of oral-based sensor technologies. The use of such sensing systems in, e.g., clinical and sports settings could provide multiple potential benefits. The fast improvement of microtechnology in recent years combined with the interest in data science and artificial intelligence opens possibilities for sophisticated uses of biological and physical signals that are measurable in the mouth (e.g., image sensors combined with classification algorithms to detect oral pathologies, or inertial sensors combined with artificial intelligence to classify head impacts).

### 4.1. Summary of Evidence

The number of papers, study designs and sensor technologies vary widely across the fields of study.

As expected, dentistry represents the majority of the papers with studies investigating bite force, soft tissue, implant stability, and oral pathologies. Dentistry also has the earliest reports of intraoral sensors development, with 37% of the identified studies published before 2010. Later studies focused more on the assessment of dental health, as well as the detection and diagnosis of pathologies, such as caries, cancer, and bruxism. Future studies could include a combination of sensing technologies and machine learning algorithms to further expand on the classification and characterization of oral diseases.

Neurology is the second field of study with most papers exploring intraoral sensors. All the identified studies in this field were published after the year 2000. The research has mainly explored the miniaturization of sensor technology and the advancements in computer capabilities to facilitate the diagnostic of neurological disorders using the oral cavity. The field has extensively applied machine learning algorithms for data cleaning and pathology characterization. However, multisensorial approaches and establishing a commonly accepted medical reference of certain conditions (such as traumatic brain injury) are recommended. This should reduce biases and provides a more clinical generalisable assessment of the technologies.

In a similar trend, the field of physical medicine and rehabilitation is rather new to this area with all papers found being published after 2010. There is a research focus on the potential that the oral cavity might bring in improving the wellbeing of paralyzed individuals and athletes. As the application of sensor technologies enables objective measurement of athletic performance and fitness, more applications of intraoral sensors for non-clinical scenarios are expected to appear. This will likely become a growing area of research interest.

The three other fields of the study found were internal medicine, obstetrics, and aerospace medicine. It shows that the sensing within the oral cavity scales across a variety of bio(medical) fields, even those usually dominated by sensor technologies that explore other body parts.

On the sensor development frontier, the most used sensors are force and pressure sensors, possibly due to the early interest in bite forces. More recently, force sensors have been used to properly quantify tongue strength, biting and deglutition patterns of individuals [42,44,45,116,117]. The use of force sensors combined with machine learning techniques enables the design of assistive technologies that aim to improve the quality of life of patients [116].

The most recent sensor advancements were focused on optical or image sensors, despite the likelihood that radiographic technologies could be under-reported in this review. Due to safety issues related to ionizing radiation, most of the digital intraoral radiology sensors are not tested in vivo and therefore did not qualify for this review. Indeed, even though some papers report strong accuracy and agreement metrics, they are frequently only tested in extracted teeth or cadavers [120,121,122]. Other reviews explore intraoral radiology in more detail [123,124]. With advancements in image processing algorithms, imaging devices are ideally placed to leverage these developments to further improve the image analsyis pipeline and provide automatic detection or diagnosis of medical conditions.

Additionally, on the rise is the use of inertial sensors in the mouth for the detection of head impacts. It is noticeable that similar methodologies for the evaluation of the devices across several research groups have been applied, which indicated good standardization in this area of research. Based on the studies found, there are however still technical barriers to using sensor data to accurately monitor head impacts and relating the data with concussions. These mainly relate to the accurate representation of head injury and the current data modalities that are being measured. On a technological level, future studies may focus on combining the data from inertial sensors with other sensor technologies to further understand the physiological outcomes of head injuries.

This review also observed some papers highlighting the difficulties in the oral environment. For temperature sensors, besides the moist environment, the mouth poses challenges due to the wide temperature variation of the oral cavity. Indeed, the studies observed temperature variations in the oral cavity between 25.9 and 43.5 °C [79]. This study also observed different temperature variations depending on the location in the oral cavity. These results have great implications for the monitoring of body temperature through the mouth. Some of the identified studies applied electronic thermometers under the tongue in sublingual pockets. These measures were taken following the standard medical practice to reduce the effect of mouth breathing and saliva evaporation, which are known to change the temperature in the mouth. However, this practice may not be viable for sensors that are kept in the mouth for long periods. Especially if they are meant to be used during for example physical activity. Future studies could investigate appropriate sensor placements, sensitivities, and response times to mitigate the challenges of measuring temperatures in the oral cavity.

Only one study employing a biochemical sensor in the oral cavity was found. Although recent studies suggest there is great potential for the use of intraoral biomarkers in health monitoring, most of the recent advancements were evaluated in laboratory bench and animal tests. They were not yet focused on reporting the accuracy and agreement of the sensors in more real-world conditions. The challenges remain in how to best deal with temperature variation, the ingestion of food and liquids, and the variability of the salivation rate. These are likely barriers to the use of biochemical sensors in the human oral cavity and need to be addressed.

Other sensors used in the mouth were microphones. One study suggests that intraoral breathing audio has a higher signal-to-noise ratio than tracheal sensors, and another study demonstrated the potential use of intraoral breathing audio to estimate perceived exertion. As could be said for research topics with few studies, this may indicate that issues related to usability and technological challenges are great barriers to the use of intraoral sensors. Nevertheless, oral microphones offer a great opportunity for monitoring athletic performance, if technical challenges related to data processing are addressed.

One interesting recent advancement was the use of bioelectrical sensors to assess tongue properties and facilitate the diagnostic of speech and swallowing disorders and rehabilitate tongue motor control in individuals with neurotraumas. All the studies with this technology were published after 2020, which demonstrates a recent maturity in the technology. Forthcoming studies in bioelectrical sensors should further investigate the clinical relevance of measured signals, especially tongue impedance.

Within the studies analysed, the area of implant stability sensors demands further investigation. A vibrating magnetic system allows clinicians to predict the survival rate and diagnose bone adhesion of teeth and implants. Although this review found three papers reporting the reliability of the measurement system, all three studies were published by the same research group, had similar methods, a possible overlap of the sampled population and came to the same conclusion from their findings [68,69,70]. The results from these studies should be reviewed with care since it is unclear how generalizable the findings are. More studies exploring the use of the resonance frequency analysis technique in different populations and different study designs would strengthen the evidence available.

The application of oral sensors is linked to specific clinical problems. It is therefore important to develop sensing devices that are traceable, and the development pipeline should include planning and testing to ensure reliability, safety, and stability requirements are met. Meeting these requirements allows a manufacturer to bring this medical technology to market. Medical devices cannot reach the market without clear evidence in terms of safety and performance. For this reason, MedTech development often uses a development road map based on the V Model [125] with some iterative cycles to allow for agile development techniques to be integrated [126].

**Figure 4:**
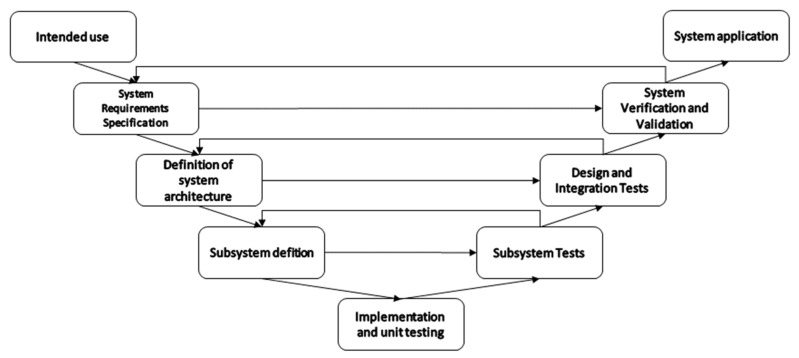
Graphical representation of a V-Model development road map of measuring systems.

As illustrated in Figure 4, the V model structure accommodates the generation of documentation and requirements planning, while agile techniques can be integrated to cater for the variability of the development. The system development starts with the definition of the intended use. At this stage, the scope and application are established through discussions with stakeholders. Next comes the stage of “system requirements specification”. During this step, the details of the clinic use, functionalities, control, performance, size, etc are defined. The third step consist of defining the system architecture and decomposing the system into subsystems. Subdivisions can consist of hardware, data handling, data presentation, etc. The fourth step is the subsystem definition, where appropriate technical solutions are chosen for each subsystem, such as the sensor technology, resolution, range, sensitivity, response times, encapsulation, placement, power, communication, and so forth. The fifth step combines implementation and unit testing. In this step, unit tests are used to ensure early verification of specific components of the hardware and software. It is also used for the quantification of some non-functional performance parameters for each component. Following the implementation, a series of test phases ensures that the systems, subsystems, and all main modules work according to the needs and requirements that have been established in the previous phases. Subsystem tests verify that each separate subsystem has the desired behaviour and output once stimulated by know signals. The design and integration tests verify that all the system modules and subsystems behave as desired once integrated with other subsystems. The system validation and verification tests ensure that the system can be applied in the desired setting. It should show that the system is effective in the target application. Finally, once all the development phases are completed, the system should be able to be applied as intended.

As this review focused on accuracy and agreement established during application within living humans, most of the captured studies report on tests that are considered in the later phases of the V-model. This can also be observed in the information given in Table 2. The considerations only include notes that are related to the stage at which a certain technology is tested. Some information that is relevant at the unit testing, for example, phase might not be captured, as this is not something that is frequently assessed alongside system verification and validation.

### 4.2. Limitations

Several challenges and limitations were identified in the synthesis of this review. A publication bias is identified due to the choice of searching only peer-reviewed journal articles. This choice limited the identification of early conference papers reporting new technological advancements or commercially driven projects resulting in an overall low number of representative reports of each area of study and sensor technology. The choice, however, was taken to facilitate finding high-quality reports with more meaningful data from potentially more mature fields of study.

The inclusion criteria accepting only measurements of accuracy and agreement and rejecting papers mentioning only correlation or other insufficient performance metrics also greatly limited the potential for finding new studies addressing other research fields or some of the challenges observed in the identified fields. The inclusion choice was taken to enable finding studies with concrete impactful results. However, until research on a certain technology reaches maturity to allow for agreement and accuracy metrics to be carefully assessed there is potential for under-reporting and lead-time bias.

The inclusion criteria also meant that studies focused on later stages of the development, resulting in reports with limited information on technical details of each sensor technology, whilst more information was included with regard to the validation of the systems during real-world applications.

## 5. Conclusions

Oral-cavity sensor systems are mostly researched in dentistry and neurology. Specific applications have already reached a certain level of technological maturity, such as the measurement of bite strength and forces on teeth. However, other sensing technologies are lacking behind and promising developments in, e.g., biochemical sensors still require further translational research. A key source of error identified for oral cavity sensing is temperature variations, this factor will impact the measurement outcomes of many sensors. In particular, the levels of accuracy and agreement obtained in a laboratory environment might not be representative of performance in the real world. New research should be encouraged to employ robust performance metrics to evaluate their systems and to increase real-world data collection.

## Figures and Tables

**Table 1:** Quality Assessment Checklist.

Item	Description	Papers (#/%)
1	Does the study address a clearly focused question?	72/88%
2	Do the authors discuss how they decided which method to use?	68/83%
3	Is there sufficient detail on the methods used?	69/84%
4	Is the method of data collection well described?	68/83%
5	Was there a comparison with an appropriate reference standard?	46/56%
6	Are the results presented in a complete and comprehensible way?	67/82%
7	Are we sure about the results, consequences and cost of alternatives performed?	66/80%
8	Can the results be applied to the population of interest?	70/85%
9	Can the method be applied to the population of interest?	79/96%
10	Are the explanations for the results plausible and coherent?	75/91%
11	Are the results of the study compared with those from other studies?	71/87%
12	Did the authors identify any limitations?	63/77%
13	Was ethical approval sought?	59/72%

**Table 2:** A list of field of application for each sensor technology, as well as their performance metric and any technical consideration reported on within the papers. It should be noted that a single paper or study might explore multiple sensor technologies.

Force/Pressure Sensors				
Technology	Papers (#/%)	Applications	Performance Metrics	Technical Considerations
Strain gauge-based	13/15%	Measuring bite force, mechanical pain threshold, lateral forces on teeth, oro-lingual pressures, chewing efficiency, detection of swallow	Calibration error, measurement error, accuracy, true positive rate and false positive rate, intraclass correlation coefficient, kappa analysis	Readings affected by sensors size [33,34], encapsulation [34], response time [35], measurement direction [35,36], quantity [36], and placement [36], also by oral temperature variation [33,37] and subjects’ anatomy [38]
Piezoelectrical	4/5%	Measuring bite force, forces between a laryngoscope and soft tissue during surgery, oro-lingual pressures, diagnose sleep bruxism	Accuracy, F1-score, sensitivity, specificity, positive predictability, negative predictability, calibration error, dynamic validation error, measurement drift error, standard error of measurement, minimal detectable change, intraclass correlation coefficient	Readings affected by sensor size [39], output variation [40], measuring direction [41], quantity, placement [40,42] and loading hysteresis [40] as also by oral temperature variation [42] and subjects’ anatomy [40]
Diaphragm	5/6%	Measuring bite force, lateral forces on teeth, oro-lingual pressures	Calibration error, intraclass correlation coefficient, method error, Cronbach’s alpha	Readings affected by sensor connections [43], placement [42], and size [43], as also by oral temperature variation [37,42,43]. Sensors affect subjects [42,43]. Sensors are adequate to measure soft tissue contact and bolus pressure [44,45]
Force sensing resistor	2/2%	Measuring bite force, diagnose dysphagia in stroke patients	Intraclass correlation coefficient, sensitivity, specificity	Readings affected by non-linearity and load rate-dependent properties of sensor [46]
Hydraulic	2/2%	Measuring bite force	Intraclass correlation coefficient	The bite element is soft, improving safety and comfort for recording bite force [47,48]
Polymer composite	1/1%	Diagnose sleep bruxism	Sensitivity, specificity	Sensor size and oral liquids are key design factors. Sensor’s polymer hysteresis and sensitivity limit measurement of actual force [49]
Capacitive	1/1%	Classify oral hypofunction	Sensitivity, specificity	Sensor thickness is a key design factor [50]
**Optical/Image Sensors**				
Photoplethysmography	5/6%	Measuring blood SpO2, diagnose dental pulp vitality	Accuracy, sensitivity, specificity, negative predictive value, positive predictive value.	Readings affected by background absorption due to venous blood and tissue constituents [51], sensor type and holding probe [51,52,53,54,55], as well as age, gender, and general health of the patient [52]
X-ray radiography	6/7%	Assess dental health, measure external root resorption, diagnose alveolar bone defect and caries	Percentage error, absolute error, kappa analysis, Cronbach’s alpha, intraclass correlation coefficient	Readings can be affected by sensor technology [56,57], configuration [58,59], size [57,60], and positioning [61]. Examiner experience [61], and definition of diagnostically acceptable images [56] and absolute ground truth [58] can bias results
Near-infrared spectroscopy	3/4%	Measuring blood SpO2, diagnose caries	Root mean squared error	Readings affected by probe fixation [62], motion artefacts [62] and saliva accumulation [62]. Definition of diagnostically acceptable images and absolute ground truth [63], examiner experience [57], and access to intact proximal surfaces [63] can bias results
Resonance Raman spectroscopy	1/1%	Measuring tissue blood SpO2	Clarke Error Grid, kappa analysis	Readings affected by probe fixation, motion artefacts and saliva accumulation. Comparison with diagnostically acceptable ground truth is difficult [64]
Infrared camera	1/1%	Diagnose subsurface cancer	Accuracy	Readings affected by temperature, moist, airflow and sensor stability [65]
Endoscope camera	1/1%	Human–computer interface for severely paralyzed individuals	Percentage error	Readings affected by environmental light intensity, system cables and size [66]
**Magnetic Sensors**				
Mechanical resonance analysis	5/6%	Measuring implant stability, predict failure of implants, diagnose dental ankylosis	Intraclass correlation coefficient, sensitivity, specificity	Readings affected by anatomical site [67], implant system [67,68,69,70], time between assessments [67], and dental damage [71]
Magnetic field sensors	5/6%	Human–computer interface for severely paralyzed individuals, measuring chewing force, and tooth displacement.	Accuracy, root mean squared error, calibration error	Readings affected by head movement [72], shapes of magnets [72], arrangement of sensors [72,73,74,75], tooth contact [72], oral temperature variation [72,73], face anatomy [72], and kinematics measurement [76]
**Temperature sensors**				
Silicon-based	4/5%	Measuring oral appliance use time, intraoral temperature	Intraclass correlation coefficient and Bland–Altman analysis	Readings affected by embedding material [77,78], sensor dimensions and placement [77], measuring setting [77,78,79,80], oral temperature variation [77,78,79,80], air flow [77,78,79,80], humidity [77,78,79,80], and time to steady-state reading [78].
Thermocouple	3/4%	Measuring body temperature, temperature changes in teeth	Bland–Altman analysis and mean error, calibration error	Readings affected by application setting [81,82,83], air flow [81,82], oral temperature variation [81,82,83], humidity, and sensor size [83]
Thermistor	2/2%	Measuring body temperature	Bland–Altman analysis	Readings affected by application setting [81], air flow [81,82], oral temperature variation [81,82], and sensor encapsulation [84]
Heat-sensitive plastic	1/1%	Measuring body temperature	Bland–Altman analysis	Readings affected by application setting, oral temperature variation [82]
**Inertial Sensors**				
MEMS accelerometer and gyroscope	8/9%	Identify and measure head impacts	Accuracy, Sensitivity, specificity, true positive rate, true negative rate, F1 score, concordance correlation coefficient, positive predictive rate, per cent and normalized root mean squared error of bench test.	Readings affected by sensing threshold [85,86], mandible movements and variations [85,87,88,89], saliva [90], and remotion of mouthguards from the mouth [86]. Video validation is time-consuming and biased by reviewers [85,86,88,91], camera angles [91], image quality [89,91] and sports dynamics [88,90,91]
**Sonography**				
Endosonography	6/7%	Assess cancer invasion, measure cancer tumour thickness, assess oral soft tissue, bone loss, abscesses, and cellulitis	Sensitivity, specificity, predictive value, Bland–Altman analysis and positive predictive value, intraclass correlation coefficient, method error, accuracy, kappa analysis	Readings affected by sensor size [92], field of exploration [93,94,95,96] and frequency [93]. Clinicians’ experience can bias results [95,97]
**Inductive sensor**				
Inductance resonating sensor	4/5%	Human–computer interface for severely paralyzed individuals	Percentage error, accuracy, root mean squared error.	Readings affected by connections [98], coil pads shape [98], coil pad layout [98,99,100], activation method [101], involuntary activations [99], oral temperature variation [99]
**Bioelectrical sensors**				
Bioimpedance	1/1%	Assess the conduction properties of the tongue	Accuracy	Readings affected by electrode polarization impedance. Needs validation as a diagnostic biomarker [102]
Electromyography	1/1%	Assess oral-cavity and oropharyngeal muscles for neuromuscular pathology	Kappa analysis	Readings affected by access to subcutaneous muscles, movement, and comfort [103]
Electro tactile	1/1%	Rehabilitation of tongue motor control	Error score	Readings affected by sensor size and placement, tongue impedance and patient and sensor sensitivity to stimulation [104]
**Microphone**				
MEMS	2/2%	Diagnose obstructive sleep apnea, estimate perceived exertion	True positives, false positives, false negatives, root-mean-squared error, normalised root-mean-squared error	Intraoral breathing audio may have a higher signal-to-noise ratio than tracheal sensors [105]. Coughing, speech, movement noise and spurious breathing events need to be filtered out [106].
**Biochemical sensor**				
pH sensor	1/1%	Measuring salivary pH	Calibration error	Long-term measurement is challenging [107]

## Data Availability

Not applicable.

## Supplementary Materials

The following supporting information can be downloaded at: https://www.mdpi.com/article/10.3390/s23020588/s1, Table S1: Summary of Dentistry Study Characteristics; Table S2: Summary of Neurology Study Characteristics; Table S3: Summary of Physical Medicine and Rehabilitation Study Characteristics; Table S4: Summary of Internal Medicine Study Characteristics; Table S5: Summary of Obstetrics Study Characteristics; Table S6: Summary of Aerospace Medicine Study Characteristics.
