# Supplementary material for: Performance of Oral Cavity Sensors: A Systematic Review
Source: Sensors (Basel). 2023 Jan 4;23(2):588. doi: 10.3390/s23020588 (PMC9862524; doi:10.3390/s23020588)
Supplement: Supplementary file 1 [file sensors-23-00588-s001.zip › Table S1 - Summary of Dentistry Studies.pdf]

TABLE S1

SUMMARY OF DENTISTRY STUDY CHARACTERISTICS SORTED BY RESEARCH TOPIC, SENSOR TYPE AND AUTHOR

Author, year	Research topic	Application	Sensor Technology	Technical Approach	Evaluation	Limitations
Gopikrishna 2007 [54]	Endodontics	Diagnose dental pulp vitality	A photoplethysmograph-based multisite oxygen sensor (4 × 5 mm) attached to the tooth with a custom-made dental probe holder.	Compared diagnostic of teeth pulp vitality from oximeter and direct visual inspection of the pulp chamber.	Metric: Sensitivity, specificity, negative predictive value, positive predictive value. Subjects: 80 adult patients.	No limitations were reported.
Janani 2020a [51]	Endodontics	Diagnose dental pulp vitality	A photoplethysmograph-based dental oxygen sensor attached to the tooth with a 3D-printed dental probe holder.	Compared diagnostic of pulp vitality from the oximeter, thermal and electric tests against direct visual inspection.	Metric: Sensitivity, specificity, negative predictive value, positive predictive value. Subjects: 79 adult patients.	Background absorption due to venous blood and tissue constituents might affect the interpretation of the result.
Janani 2020b [52]	Endodontics	Diagnose dental pulp vitality	A photoplethysmograph-based dental oxygen sensor attached to the tooth with a 3D-printed dental probe holder.	Compared diagnostic of teeth pulp vitality from oximeter and direct visual inspection of the pulp chamber.	Metric: Sensitivity, specificity, negative predictive value, positive predictive value. Subjects:37 adult patients.	Sensor type and holding probe, age and gender of the patient, and general systemic conditions influence the results.
Pozzobon 2011 [53]	Endodontics	Diagnose dental pulp vitality	A photoplethysmograph-based pulse oximeter sensor with a stainless-steel dental holder (19 × 19.4 × 9mm) positioned on the tooth's buccal and palatal surfaces.	Compared diagnostic of teeth pulp vitality from oximeter and direct visual inspection treated teeth.	Metric: Accuracy rate. Subjects: 84 adult patients.	No limitations were reported.
Bhowmik 2022	Oral medicine	Diagnose subsurface cancer	A data acquisition unit with an infrared camera (wavelength 8 to 14 um) and a humidity sensor. The camera had thermal sensitivity < 50mK and a sample rate 8.7Hz. The data acquisition unit was covered with a glass lid (2.6 x 3.4 cm) and connected to a processing unit.	Compared findings from machine learning algorithm with intraoperative and histological findings.	Metric: Accuracy rate. Participants: 8 volunteers from a mixed population.	No limitations were reported.
Heppt 1993 [93]	Oral medicine	Assessment of cancer invasion in the mandible	Two flexible, digitally guided ultrasound transducers. One of 5MHz and the other of 7.5MHz. Transducers were attached to a sonographic computer.	Compared findings from ultrasounds with intraoperative and histological findings.	Metric: Sensitivity, specificity, and predictive value. Subjects: 33 adult patients.	Involvement of the cortical bone or of the periosteum result in false findings.
Yesuratnam 2014 [96]	Oral medicine	Measure tongue cancer tumor thickness	A high-resolution 15–7 MHz linear ultrasound 'hockey stick' probe. The probe had a 23 mm rectangular field of view and a single near-field focal zone. The probe was in a sterile cover with gel.	Compared measurements of tumour thickness from intraoral ultrasound and MRI against histological findings.	Metric: Bland-Altman analysis and positive predictive value. Subjects: 88 adult patients.	Images obtained varied. Not all radiological and clinical findings had resection of the lesion and nodal dissection.
Watanabe 1999 [107]	Oral pathology	Measure salivary pH	A silicone-covered denture with an iridium-oxide pH sensor at the lingual side, a reference polar electrode and a radio transmitter. The reference electrode was positioned to touch the gingiva via a ceramic interface.	Calibrated pH sensor using a 2-point calibration method and tested for 7.5h with a woman experienced with denture.	Metric: Percentage error of calibration. Subjects: 1 denture wearer.	Long-term measurement on several subjects is required to analyze and characterize patterns of pH throughout the day.

Author, year	Research topic	Application	Sensor Technology	Technical Approach	Evaluation	Limitations
Mcauliffe 2015 [49]	Oral pathology	Diagnose sleep bruxism	Microcontroller circuit boards with carbon–polymer composite pressure sensors (7.5 × 4.5 × 0.6mm) integrated into a hard acrylic bite guard.	Compared diagnostic of stereotyped bruxism and non-bruxism movements against the specialist.	Metric: Sensitivity and specificity. Subjects: 1 healthy volunteer.	It is difficult to eliminate bias or error of the examiners. The data set was small. The sensor is only for events because of polymer hysteresis and sensitivity limit measurement of actual force.
O'Hare 2022 [39]	Oral pathology	Diagnose sleep bruxism	Retainer with Bluetooth and a MEMS piezo-resistive pressure sensor. Electronics were powered by 20 mAh and a 3.7 V battery.	Compared diagnostic of stereotyped bruxism and non-bruxism movements against surface EMG.	Metric: Accuracy, f1-score, sensitivity, specificity, positive predictability, and negative predictability. Subjects: 8 volunteers from a mixed population.	Simulated bruxism activities in awake subjects. The data set was small. The device needs miniaturization.
Aziman 2019 [56]	Oral radiology	Assess dental health	A CCD-based and a CMOS-based intraoral radiography devices.	Radiologists and dentists scored pairs of images from radiography devices	Metric: Intraclass correlation coefficient. Subjects: 5 adult patients.	Analysis can only be used with diagnostically acceptable images.
Dündar 2020 [57]	Oral radiology	Diagnose caries	Four intraoral imaging sensors: an X-ray machine with Storage Phosphor Plate sensors, a near-infrared light transillumination device, an LED-based device, and a laser fluorescence device.	Compared diagnostic using each sensor technology against visual examination.	Metric: Sensitivity and specificity for technique, Cohen's kappa test for interobserver agreement. Subjects: 34 adult patients.	Only utilized a single examiner.
Elkhateeb 2022 [59]	Oral radiology	Assess dental health	Laser scanners and an intraoral X-ray machine with 7 mA 65-kVP using a photostimulable phosphor system.	Radiographs were screened by two oral and maxillofacial radiologists for evaluation of artefact type and cause	Metric: Kappa analyses, error rate. Subjects: undefined	No limitations were reported.
Metzger 2022 [63]	Oral radiology	Detection of proximal caries	Reflected near-infrared (850 nm) light radiography.	Scans of maxillary and mandibular arches using the scanner and compared to bitewing radiology in detecting proximal caries.	Metric: Sensitivity, specificity, accuracy and Kappa coefficients. Subjects: 100 adult patients.	No standardization of equipment nor absolute ground truth. Intact proximal surfaces cannot be verified.
Moghadam 2017 [60]	Oral radiology	Diagnose of bone defect in patients with periodontitis	Digital intraoral photo stimulated phosphor plate radiography sensor. The sensor had standard size 2 with a ring film holder. The sensor was positioned using a parallel technique.	Compared length from the cementoenamel junction to the alveolar margin bone was measured by Williams periodontal probe and radiography.	Metric: Intraclass correlation coefficient. Subjects: 52 adult patients.	No limitations were reported.
Natto 2022 [61]	Oral radiology	Detecting approximal caries and interdental bone loss	Digital intraoral bitewing radiograph and an X-ray sensor plate.	Horizontal and vertical bitewing radiographs for the same area were evaluated for caries by two specialists and compared to clinical examination.	Metric: Intraclass correlation coefficient and Cronbach's alpha. Subjects: 20 adult patients	The difficulty of blinding the X-rays being evaluated. Exposure was done by interns with limited experience.
Taravati 2022 [58]	Oral radiology	Measurement of the external root resorption	Digital intraoral photostimulated phosphor plate radiography sensor with 70 kvp, 0.125 s, 200 mA/s, size 22 × 35 mm, ISO 0, speed E, X-ray machine.	Root length and root resorption rates from periapical radiographs taken by specialists were assessed by 3 observers and compared to the analysis of endodontic files by two specialists blinded to the results of radiographs.	Metric: Cohen's kappa and Cronbach's alpha. percentage errors, and absolute errors.	Analysis of endodontic files is not as accurate as histological examinations.

Author, year	Research topic	Application	Sensor Technology	Technical Approach	Evaluation	Limitations
Ponukumati 2020 [40]	Oral surgery	Measure force between laryngoscope and soft tissue during surgery	Durable plastic 3D printed laryngoscope cover (10 × 80 × 0.5 mm) and a maxillary guard (5 mm thick). The laryngoscope cover had a 0.75 mm thick sensor base with thirteen 11.3 kg piezoresistive sensors. The maxillary guard had a sensor base with three 45.4 kg piezoresistive sensors. Tegaderm film encased all sensors.	Calibrated sensors on a custom machine. Tested dynamic loading and unloading and drift due to long-term loading. Tested usability in cadavers. Performed measurements in human subjects.	Metric: Percentage error of calibration, bench dynamic drift, bench loading drift. Subjects: 3 adult patients.	Sensors' output variation and spacing affect readings. Peak forces on the maxilla were above calibrated range. Larger mandibles and heads demand a broader and denser sensor array.
Bonakdarchian 2009 [33]	Orthodontics	Measure bite force	An 8.6 mm thick stainless-steel transducer with temperature-compensated strain gauges. The transducer had a U-shape and was protected by 2mm thick plastic pads.	Calibrated strain gauge transducer and performed several measures between molars	Metric: Percentage error. Subjects: 40 healthy volunteers.	Small sample size.
Castroflorio 2006 [112]	Orthodontics	Measure bite force	An inferior splint with a slot for a cylindrical compressive-force sensor (10.6 × 5mm) and a superior splint with a stainless-steel cursor.	Calibrated transducer and recorded 30 seconds of isometric contractions at 80% of the maximal force	Metric: Intraclass correlation coefficient. Subjects: 9 healthy volunteers.	No limitations were reported.
Fernandes 2003 [46]	Orthodontics	Measure bite force	A force sensor (7.5 mm thick) with a steel plate with a bulge. A circular conductive polymer pressure-sensing resistor insulated in thermoplastic. The sensor was bonded to a steel support plate using a laminating adhesive and covered with silicone dental impression material.	Compared reliability of bite force measurement of force sensing resistors against strain gauge bite fork on healthy subjects. Test force sensing resistors with denture subjects.	Metric: Intraclass correlation coefficient and method error. Subjects: 28 volunteers from a mixed population.	Nonlinear and load rate-dependent properties of the sensor.
Ferrario 2004 [34]	Orthodontics	Measure bite forces on healthy single teeth	A stainless-steel strain gauge bite force transducer (5 × 7 × 4 mm) covered in a polytetrafluorethylene band. The transducer was covered with a cap and a disposable latex sheath. The final thickness was 8.5mm.	Calibrated transducer with calibration machine and performed sequential measurements of maximum bite force at each tooth.	Metric: Percentage error of calibration and repeatability error. Subjects: 52 healthy volunteers.	No limitations were reported.
Gibbs 2002 [38]	Orthodontics	Measure bite force	Transducer with two stainless steel plates separated by a steel sphere. Four strain gauges were parallelly mounted on one of the plates and covered by acrylic resin.	Calibrated strain gauge-based transducer with calibration machine and performed measurements of maximum bite force.	Metric: Percentage error of calibration. Subjects: 44 adult patients.	Some subjects' masseter muscles were narrow and produced little bulge, indicating low muscle strength.
Iwasaki 2022 [50]	Orthodontics	Classify oral hypofunction	Capacitive pressure-mapping sensor. The sensor had a dielectric layer sandwiched between two electrode layers one with 7 and the other with 9 electrodes.	Functional teeth identification from maximal clenching in the intercuspal position was compared between different sensors against clinical analysis	Metric: Sensitivity and specificity. Subjects: 596 adult patients.	Only had subjects from a specific area. Findings may not be applicable to frailer individuals. Did not test agreement for measurements.
Lear 1965 [35]	Orthodontics	Measure lateral forces on teeth	Three stainless steel laminates separated by welded spacers forming a 1.5mm thick Z-shaped transducer. Two strain gauges are cemented parallelly on the central laminate. The movement of wires is prevented by an eyelet.	Calibrated transducer with calibration machine and tested variations when person talks, keeps the mouth closed, and apply forces to different parts of the transducer.	Metric: Percentage error of calibration. Subjects: 5 healthy volunteers.	The system may not read sudden high forces. Lateral movement of the teeth may affect the reading. Transducer thickness is the largest source of errors.
Levartovsky 2022 [47]	Orthodontics	Measure bite force on subjects with implanted	A 17 x 5.4 x 63.5 mm bite fork device with a hydraulic pressure gauge covered with a diaphragm. The disposable vinyl biting element	Use factory calibrated sensor and measured the maximum clench in three occluding pairs	Metric: Intraclass correlation coefficient. Subjects: 53 Adult patients.	Small study population. No measurements were performed before the implants were

Author, year	Research topic	Application	Sensor Technology	Technical Approach	Evaluation	Limitations
		teeth	was encased in a polyethene tube.	on each posterior semi arch.		rehabilitated
Luffingham 1968 [43]	Orthodontics	Measure soft-tissue pressure on teeth	A Plano-hemispherical (diameter 5 mm, 1.5 mm thick) acrylic switch covered with a rubber diaphragm. The switch was sealed in latex. The diaphragm activated electrodes that control a circuit measuring pressure on the switch.	Calibrated transducer switch against manometer and test temperature variation in water bath and response time with a rubber diaphragm.	Metric: Percentage error of calibration. Subjects: 50 volunteers.	The wire is thicker and shorter than that for a strain gauge. Subjects can notice the sensor's vibration.
Mansour 1977 [41]	Orthodontics	Measure bite force	Piezo electrical transducer made of natural quartz crystal discs 1.5 mm thick and varying diameters (2-6 mm). The discs were plated with chrome and gold and soldered to insulated leads. Discs were connected to an electrometer. Devices were encapsulated by two 0.5 mm layers of polyethene.	Calibrated piezoelectrical transducer with calibration machine and performed measurements of maximum bite force in different teeth.	Metric: Percentage error of calibration. Subjects: 6 healthy volunteers.	No limitations were reported.
Mokhtar 2022 [111]	Orthodontics	Measure bite force and chewing efficiency in ankylosis cases	A button-type compression transducer. The transducer had a strain gauge and could measure loads of up to 5000 N.	Use factory calibrated sensor and measured three times the maximum clenching force.	Metric: Percentage error of calibration. Subjects: 30 Adult patients.	No limitations were reported.
Mountain 2011 [113]	Orthodontics	Measure bite force	Unspecified single-tooth bite force gauge.	Calibrated unspecified bite sensor prong with calibration machine and performed measurements of maximum bite force.	Metric: Bland-Altman analysis and mean error. Subjects: 205 healthy children.	No limitations were reported.
Patil 2022 [109]	Orthodontics	Measure bite force	An electromechanical device which measures the mechanical deflections in the jaws.	Use factory calibrated sensor and measured three times the maximum clenching force on both sides alternating tested and reference devices.	Metric: Cohen's kappa and percentage error. Subjects: 120 volunteers from a mixed population	No limitations were reported.
Shellhart 1996 [37]	Orthodontics	Measure lip pressure on teeth	Two pressure sensors. A commercial diaphragm-type pressure transducer (input voltage of 0.9V) and a strain-gauge-based beam sensor. The beam sensor had a cylindrical plastic sensing surface (2.0 × 0.5mm). Both sensors were glued to a stent.	Calibrated beam sensor with weights and diaphragm sensor in a heated pressure chamber with an in-line pressure sensor. Measured lip pressure using a stent with both sensors.	Metric: Intraclass correlation coefficient and method error. Subjects: 22 healthy volunteers.	No limitations were reported.
Woodford 2021 [109]	Orthodontics	Measure 3D jaw kinematics and occlusal loading	System samples position and orientation of two electromagnetic field sensors (dimensions 1 × 4 × 2 mm without coating, 4 × 9 × 3 mm with coating). Sensors were submitted to a pre-defined magnetic field volume of approximately 220 cm^3 in size.	Compared occlusal loading model readings from the electromagnetic sensor when biting soft polyurethane rubber sample with compression force on the rubber sample from universal testing machine.	Metric: Root-mean-squared error. Subjects: One healthy adult.	Small sample size. errors in kinematics measurement translate to variability in occlusal loading. Material model, contact formulation, mesh discretization and the solver used in the finite element analysis can introduce errors.
Yamada 1990 [72]	Orthodontics	Measure tooth displacement	Appliance with four epoxy-insulated hall sensors (4.5 × 4.5 × 2.0 mm). Sensors were placed in a rectangle (20 × 18mm) on an acrylic sheet (25 × 30	Calibrated system using custom machine moving magnet in a square with controlled steps, tested for thermal drift	Metric: Percentage error of calibration. Subjects: 5 healthy volunteers.	Earth's magnetic field affected readings due to head movement. Shapes of the magnet, tooth

Author, year	Research topic	Application	Sensor Technology	Technical Approach	Evaluation	Limitations
			× 2 mm). A cylindrical samarium cobalt magnet (3 × 2 mm) was attached to a tooth by a plastic rod and placed within the sensor rectangle.	and performed measurements in volunteers		contact, and of face bow generate errors.
Yoshida 2000 [74]	Orthodontics	Measure tooth displacement	Two cubes (1 mm^3) with eight epoxy-insulated hall sensors (2.7 × 2.35 × 0.95 mm). A cylindrical neodymium magnet (4.0×1.2mm) was connected to a tooth (front and back) by plastic rods and placed within the sensor cube.	Calibrated system using custom machine moving magnets in 3D with controlled steps and performed measurements in volunteers	Metric: Percentage error of calibration. Subjects: 2 healthy volunteers.	No limitations were reported.
Yoshida 2001 [73]	Orthodontics	Measure tooth displacement	Cube (1 mm^3), with eight epoxy-insulated hall sensors (2.7 × 2.35 × 0.95mm). A cylindrical neodymium magnet (4.0 × 1.2mm) connected to a tooth (front and back) by plastic rods and placed within the sensor cube.	Calibrated system using machine moving magnet in 3D with controlled steps, tested for thermal drift and performed measurements in volunteers	Metric: Percentage error of calibration. Subjects: 3 healthy volunteers.	No limitations were reported.
Brierley 2017 [79]	Orthodontics	Measure oral appliance use time	Two acrylic-encased thermosensitive sensors. Sensors were calibrated to ±0.1°C and had 18-month battery life. Sensors were attached to molar bands, cemented palatally and buccally.	Compared 7-days non-stop use records with thermosensitive sensors records.	Metric: Percentage error. Subjects: 5 healthy volunteers.	The study had a small sample size.
Gjerde 2018 [80]	Orthodontics	Measure oral appliance use time	A commercial mandibular advancement device with a thermosensitive microsensor (10.5 × 8.5 × 4 mm) and a triaxial accelerometer. Thermosensitive sensors were calibrated to ±0.1°C, with a 5-year battery life and memory storage for up to 6 months of data. The sensors were encased in acrylic.	Compared 30-day use between self-record dairies and digital records from thermosensitive microsensors.	Metric: Intraclass correlation coefficient and Bland-Altman analysis. Subjects: 80 adult patients.	There is no fully validated adherence monitoring sensor available in the market. The study had to use retrospective self-reporting as a gold standard.
Kirshenblatt 2018 [77]	Orthodontics	Measure oral appliance use time	Three acrylic-encased thermosensitive sensors. Two were sized 12 × 8 × 4.5 mm and had 18-month battery life. One was sized 10.5 × 8.5 × 4 mm and included a 24-months battery life and a triaxial accelerometer. All sensors were calibrated to ±0.1°C. Sensors were placed on the buccal area of acrylic retainers.	Test sensors in a water bath for different use times and embedding material. Compared 30-day use between self-record dairies and digital records from two sensors.	Metric: Percentage error. Subjects: 14 healthy volunteers.	Variations in embedding thickness and water temperature could impact accuracy. Some sensors were faulty. The water baths did not test the accelerometer.
Khan 2007 [48]	Periodontics	Measure bite force	A hydraulic bite fork is modified by attaching the head of a Tooth Slooth (18 × 8 × 5 mm) with adhesive.	Calibrated bite fork with calibration machine and performed several sessions of measurements of maximum bite force.	Metric: Intraclass correlation coefficient. Subjects: 44 adult patients.	Confounding factors include the effects of gender on maximal bite force.
Bertram 2008 [95]	Periodontics	Assess periimplant buccal bone loss	A linear 12.5-MHz small-part intraoral ultrasound transducer. The transducer was positioned parallel to the axis of the implants with a perpendicular projection to the implant	Compared measurements of the length between the upper thread of the implant and marginal bone from ultrasound and surgically using a calibrated periodontal probe.	Metric: Intraclass correlation coefficient and method error. Subjects: 25 adult patients.	The inadequate time interval between sessions can affect results. Misinterpretation can affect the assessment of bone defects.
Costantino 2013 [97]	Periodontics	Diagnose peritonsillar abscess or	An 8–5 MHz intracavitary ultrasound transducer.	Compared diagnosis of patients assessed using either ultrasound or needle aspiration after inspection.	Metric: Accuracy. Subjects: 28 adult patients.	Clinicians' availability and ultrasound experience may have biased studies.

Author, year	Research topic	Application	Sensor Technology	Technical Approach	Evaluation	Limitations
		cellulitis				
Fu 2022 [92]	Periodontics	Assessment of periodontal disease	A hockey stick transducer with 14 2.5-mm-diameter fibres bundled receiving photoacoustic signals. The transducer had a central frequency of 9 MHz and a bandwidth of 7-15 MHz. Fibres were coupled to a tunable optical parametric oscillator laser operating at 680–970 nm. An ultrasound data acquisition system with 256 channels and a sampling rate of 62.5 MHz collected and pre-processed photoacoustic signals.	Characterized the performance of the system with tissue-mimicking phantoms and ex vivo porcine jaws then imaged a human subject.	Metric: Intraclass correlation coefficient and Bland-Altman analysis. Subjects: 1 healthy volunteer.	The gap and the size of the fibre module prevent the transducer from covering all the teeth.
Salmon 2012 [94]	Periodontics	Assess soft-tissue	A single lead zirconate titanate ceramic transducer (3.6 mm diameter). The transducer was immersed in a coupling liquid inside a chamber (15 × 18mm). The chamber was closed by a 0.2 mm 8BK membrane. Scans used an ultrasonic gel coupling.	Compared assessment of different specialists using intraoral ultrasound in correctly positioning and identifying dental landmarks in healthy teeth.	Metric: Cohen's kappa coefficient. Subjects: 3 healthy volunteers.	The field of exploration may be low to visualize deep periodontal pockets. Difficult to explore wisdom teeth and the lingual side of mandibular incisors.
Kelsey 1976 [110]	Prosthodontics	Measure bite pressure on complete dentures	A denture with eight beryllium-copper cuplike transducers. The transducers had a diaphragm (0.102mm thick) sealed with a metal disk and epoxy cement. The diaphragm's interior had a strain gauge.	Calibrated strain gauge-based pressure transducer with known weights and measured pressures caused by masticating test foods.	Metric: Percentage error of calibration. Subjects: denture wearer.	No limitations were reported.
Lundgren 1984 [36]	Prosthodontics	Measure bite force in dentures	Cylindrical hardened steel transducers (5.2 × 5.2 mm) mounted into prosthetic appliances. Transducers had two round plates joined by a z-shaped bar with a strain gauge bridge connected to a 2V, 10 mA amplifier. The devices were protected using silicon.	Calibrated strain gauge-based transducer built into dentures with calibration machine and performed measurements of maximum bite force.	Metric: Percentage error of calibration. Subjects: 1 adult patient.	Measuring only axial forces limits the study of occlusal forces. The number and distribution of transducers affect the magnitude of forces read.
Ogata 1988 [114]	Prosthodontics	Measure lateral forces on implanted teeth	A cylindrical cantilever (3.5 × 8 mm) with a beam part (3 × 2 mm) connecting the fixed part. The beam had four strain gauges. The device was built into an attachment's gold Patrix. Wires were threaded through a vinyl tube.	Calibrated transducer with known weights and performed measurements of subjects chewing different solids.	Metric: Percentage error of calibration. Subjects: 5 adult patients.	No limitations were reported.
Atieh 2014 [67]	Prosthodontics	Predict failure of implants	A device that quantifies implant stability by measuring the frequency of vibration of a magnetic peg attached to a tooth.	Identified implant stability quotient level at 8 weeks that best reflects clinical implant survival after 1 year.	Metric: Sensitivity and specificity. Subjects: 28 adult patients.	Small sample size and follow-up period, single anatomical site and implant system.
Bertl 2012 [71]	Prosthodontics	Diagnose dental ankylosis	A device that quantifies implant stability by measuring the frequency of vibration of a magnetic peg. Peg was fixed to the primary molar using a light-cured composite.	Compare diagnostic using implant stability device against specialist using percussion method	Metric: Sensitivity and specificity. Subjects: 15 adult patients and 30 healthy volunteers.	Small sample size and the reference tests applied which required patients with high root damage.
Herrero-Climent	Prosthodontics	Measure implant stability	A device that quantifies implant stability by measuring the frequency of vibration of a	Using an implant stability device, perform several stability measurements	Metric: Intraclass correlation coefficient. Subjects: 19 adult	No limitations were reported.

Author, year	Research topic	Application	Sensor Technology	Technical Approach	Evaluation	Limitations
2012 [68]			magnetic peg. Pegs were screwed directly on the implant without the interposition of the prosthesis pillar.	on implants with two different magnetic pegs.	patients.	
Herrero-Climent 2013 [70]	Prosthodontics	Measure implant stability	A device that quantifies implant stability by measuring the frequency of vibration of a magnetic peg. Pegs were screwed directly on the implant without the interposition of the prosthesis pillar.	Using an implant stability device, perform several stability measurements on implants with two different magnetic pegs.	Metric: Intraclass correlation coefficient. Subjects: 23 adult patients.	No limitations were reported.
Jaramillo 2014 [69]	Prosthodontics	Measure implant stability	Two versions of a device that quantifies implant stability by measuring the frequency of vibration of a magnetic peg. Pegs were screwed on the implant.	Perform several measurements on each implant with two different magnetic pegs and implant stability devices.	Metric: Intraclass correlation coefficient. Subjects: 15 adult patients.	No limitations were reported.
Park 2022 [83]	Prosthodontics	Measure temperature changes in gold-restored teeth	A nickel/chromium-nickel/aluminium, K-type thermocouple sensor. The sensor has a 0.5 mm diameter and was insulated with duplex. The sensor was attached to a coronal third of the buccal surface with a 3mm flowable resin composite.	Calibrated transducer between 10-40°C in a controlled water bath. Use a sensor while participants consumed hot and cold drinks according to a standardized regimen.	Metric: Percentage error of calibration. Subjects: 10 healthy volunteers.	No limitations were reported.
