# Supplementary material for: Performance of Oral Cavity Sensors: A Systematic Review
Source: Sensors (Basel). 2023 Jan 4;23(2):588. doi: 10.3390/s23020588 (PMC9862524; doi:10.3390/s23020588)
Supplement: Supplementary file 1 [file sensors-23-00588-s001.zip › Table S2 - Summary of Neurology Studies.pdf]

TABLE S2
SUMMARY OF NEUROLOGY STUDY CHARACTERISTICS

| Author, year | Research topic | Application | Sensor Technology | Technical Approach | Evaluation | Limitations |
|---|---|---|---|---|---|---|
| Castillo 2017 [105] | Sleep disorders | Diagnose obstructive sleep apnea | A tooth microphone is integrated into the subject's oral appliance. The sampling frequency of 44.1 kHz and the 16-bit resolution. | Compared automatic detection of apnea and snores from audio recorded by an intraoral and a tracheal microphone. Validated against labels from a technician. | Metric: True positives, false positives, and false negatives. Subjects: 1 healthy volunteer. | The low number of available samples and patients. |
| Luo, 2021 [102] | Speech and swallowing disorders | Assess the conduction properties of the tongue | A printed circuit board (100 × 18 mm). with 16 contacts. Contacts were arranged in two concentric circles of radii 4 and 7 mm, respectively. Measuring directions are determined by the angles 0°,45°,90° and 150°. The board was connected to an IoT development platform powered by a 3.7 V, 2200 mAh lithium polymer battery. | Subjects rinse their mouth with a small amount of water then placed a system centred on the tongue and data collected. | Metric: Accuracy. Subjects: 7 healthy volunteers. | The maximum number of measurements in one session is limited. Tongue-electrode polarization impedance affects measurements. Small sample size. Not fully clinically validated as a diagnostic biomarker |
| Menon 2022 [103] | Speech and swallowing disorders | Assess oral-cavity and oropharyngeal muscles for neuromuscular pathology | Single-use bipolar trans membranous EMG recording device. The EMG device had two 0.75 mm diameter stainless steel electrodes located at the distal end of the probe separated by 0.75 mm. The probe cable has two 1.5 mm touch-proof connectors for each electrode and one crimp wire connector pin. The connector pin was plugged into a conventional surface ground electrode. | Two otolaryngologists positioned trans membranous EMG and needle EMG on the mucosal surface correlating to the expected midpoint of right and left palatoglossi and genioglossi. | Metric: Cohen's kappa. Subjects: 16 mixed adults. | Probe accuracy was not confirmed. The study did not include participants with myopathy and results cannot identify myogenic processes. |
| Hadley 2015 [116] | Speech and swallowing disorders | Automated detection of swallow | A custom-fit vacuum-formed PVC mouthpiece with seven pressure transducers. The transducers were miniature stainless-steel diaphragms with strain gages secured by wax. Transducers encased in PDMS. Wires were interwoven and curved behind the posterior molar with wax. | Compare the classification of swallow and non-swallow events measured by instrumented oral appliances against human labelling | Metric: Accuracy, true positive rate and false positive rate. Subjects: 19 mixed adults. | The study was all performed post hoc. A wireless mouthpiece would enable increased mobility while using the device. |
| Konaka 2010 [117] | Speech and swallowing disorders | Diagnose dysphagia in stroke patients | A 0.1 mm thick T-shaped sheet with five force sensing resistor sensors wired to a computer. The sensor sheet is attached to the palate directly with sheet-type denture adhesive. | Compare diagnostic of dysphagia by T-shaped sensor sheet against specialist assessment using the drinking water test. | Metric: Sensitivity and specificity. Subjects: 64 adult patients. | Tongue pressure cannot completely predict dysphagia. The electromyographic activity of muscles and swallowing sounds should contribute. |
| Manning 2016 [44] | Speech and swallowing disorders | Measure oro-lingual pressures | A Plano-hemispherical MEMS (16 × 3mm) with a digital pressure sensor protected by a soft protection gel. The sensor was coated with a non-stick media isolation material and encased by a soft polymer. Chip is cabled to a headset and transmission module. | Using the sensor performed several tests swallowing controlled volumes of water and custard. | Metric: Intraclass correlation coefficient and Cronbach's alpha. Subjects: 57 healthy volunteers. | Small sample size and a lack of gender balance across the age groups. Non-natural bolus delivery method (using a syringe) and swallowing to command. |

| Author, year | Research topic | Application | Sensor Technology | Technical Approach | Evaluation | Limitations |
|---|---|---|---|---|---|---|
| Martinez 2022 [118] | Speech and swallowing disorders | Measure tongue strength | A piezo-resistive sensor connected to a data acquisition system | The recording sensor was glued to the upper hard palate, immediately above the central incisor line and exactly in the middle of the anterior alveolar ridge | Metric: Intraclass correlation coefficient, standard error of measurement and a minimal detectable change | No direct comparison with a clinically accepted reference method. Results are not generalizable to other age groups. Reproducibility is limited to instant retests. Measuring direction is not functionally oriented and ecologically valid. |
| McCormack 2015 [45] | Speech and swallowing disorders | Measure oro-lingual pressures | A Plano-hemispherical MEMS (16 × 3 mm) with a digital pressure sensor protected by a soft protection gel. The sensor was coated with a non-stick media isolation material and encased by a soft polymer. Chip is cabled to a headset and transmission module. | Calibrated sensor against mercury sphygmomanometer and measured tongue strength and tongue endurance pressures of participants | Metric: Intraclass correlation coefficient. Subjects: 35 healthy volunteers. | Small sample size. |
| Searl 2003 [42] | Speech and swallowing disorders | Measure oro-lingual pressures | A diaphragm-type (2.33 × 6.51 × 0.62 mm) and a strain gauge-based (2.80 mm diameter x 0.45 mm thickness) pressure sensor. Sensors were attached to the mouth or a palatal appliance (0.5 mm thick) using a biotape. The appliance was thermoformed with acrylic. | Calibrated sensors against a manometer, tested temperature drifts using animal tissue and tested different placements in the mouth of subjects. | Metric: Percentage error of calibration. Subjects: 5 healthy volunteers. | Precise placement of the transducer with the tape was time-consuming and placement was inaccurate. The acrylic plate results in alterations in speech. |
| Gabler 2020 [85] | Traumatic brain injury | Quantify head impacts | Custom-fit mouthguards instrumented with flexible electronic boards. The electronic boards contained triaxial linear and angular accelerometers and supporting hardware protected by conformal coating and encased in protective materials. | Compare impacts identified with machine learning classification and a mouthguard instrumented with inertial sensors against impacts from video recordings labelled by humans. | Metric: Sensitivity and specificity. Subjects: 21 healthy volunteers. | Sensing threshold, impact severity, low number of recorded impacts and reviewer bias affect performance. Wearer chewing can damage the electronics. Longer battery life and durability are needed. |
| Goodin 2021 [91] | Traumatic brain injury | Quantify head impacts | A custom-fit mouthguard instrumented with three triaxial ± 200g accelerometers and a ± 2,000 dps range gyroscope. | Compare impacts identified with the instrumented mouthguard and classification method against impacts from video recording from four angles labelled by humans. | Metric: True positives, true negatives, and F1 score. Subjects: 64 healthy volunteers. | The possibility that hits were not captured during the process could have been present but not noted and some types of impact may not have been recorded. The classifier was applied to elite adult athletes with few females and may not generalize to child, adolescent, amateur or female athletes. |
| Kieffer 2020 [86] | Traumatic brain injury | Quantify head impacts | One custom-fit and two boil-and-bite mouthguards that house a triaxial accelerometer and a triaxial gyroscope. | Compare the concordance of measurements of several head impact devices including instrumented mouthguards with the anthropomorphic test device. Validated impacts identified in sports against impacts from video identified by a specialist. | Metric: Concordance correlation coefficient and positive predictive value. Subjects: Healthy volunteers. | False positives were generated when players remove mouthguards from their mouths during breaks. Sensing threshold and the number of recorded impacts affect performance. Devices were tested in different on-field conditions. |
| King 2015 [90] | Traumatic brain injury | Quantify head impacts | A moulded instrumented mouthguard containing a low power, ± 200g triaxial accelerometer and a triaxial angular rate gyroscope. | Compare measurements of the instrumented mouthguard with an anthropomorphic test device and performed data collection in rugby. | Metric: Root-mean-squared error of bench test. Subjects: 38 healthy volunteers. | Mouthguards were bulky and required customizing. Saliva troubled data download from mouthguards. Not all rugby activities were validated or could be verified. |

| Author, year | Research topic | Application | Sensor Technology | Technical Approach | Evaluation | Limitations |
|---|---|---|---|---|---|---|
| Kuo 2018 [89] | Traumatic brain injury | Quantify head impacts | Custom-fit ethylene vinyl acetate mouthguard with a triaxial accelerometer, a triaxial gyroscope and a proximity sensor to detect teeth. The device lasts 3.5 hours and collects 1638 events. | Compare impacts identified with the instrumented mouthguard against impacts from video recordings labelled by humans. | Metric: Accuracy rate. Subjects: 7 healthy volunteers. | Video assessment takes time and is affected by video quality and quantity. Lower mandible disturbances may affect measurements. The sample size and period were small. |
| Rich 2019 [88] | Traumatic brain injury | Quantify head impacts | Acrylic mouthpieces containing a ± 200 g triaxial linear accelerometer, a ± 40 rad-$s^{-1}$ triaxial gyroscope, a 3.7 V, 40 mAh lithium polymer battery and a wireless charger. The device collects up to 255 impacts and exports data through Bluetooth. | Compared measurements of the instrumented mouthguard against an anthropomorphic test device with a 3D-printed dental arch. Validated impacts identified by the mouthguard in football against impacts from video identified by specialists. | Metric: Percent and normalized root mean squared error. Sensitivity and positive predictive value. Subjects: 4 adult patients. | The sample size was small. Mandible effects, skull coupling, and a wider range of sports and activities need to be further tested. |
| Waltzman 2021 [119] | Traumatic brain injury | Quantify head impacts | Boil-and-bite mouthguards with triaxial accelerometer and gyroscope optimized for impacts between 10g and 200g. | Use instrumented mouthguard pre-validated with an anthropomorphic test device and performed data collection in flag American football and tackle football. | Metric: Normalized root-mean-squared error. Subjects: Multiple healthy adolescents | Differences from tackle versus flag football may have affected results. Head impacts were not video verified. Attendance data for the tackle football teams was not collected. Limited ability to assess higher impacts. Uneven distribution of playing and practice time among athletes. Concussion risk was not assessed. |
| Wu 2016 [87] | Traumatic brain injury | Quantify head impacts | Custom-fit mouthguard with 4 mm average thickness and 7mm height above the gum line instrumented with 6-degree-of-freedom inertial sensors. | Compared measurements of frontal head impacts of several head impact devices, including instrumented mouthguards, against high-speed video analysis based on an ear-plug reference. | Metric: Root-mean-squared, and normalized root-mean-squared errors. Subjects: 1 healthy volunteer. | Mandible motion and variations in mouthguard fabrication may affect performance. Only mild impacts were assessed. High-speed stereo video tracking was limited by ear-canal reference. |
| Soltanzadeh 2015 [55] | Vital sign monitoring | Measure blood SpO2 | A digital photoplethysmograph proto sensor attached to a brush. | Compared in a lab the blood oxygen on the four mental foramina was measured with an intraoral against a fingertip pulse oximeter | Metric: Accuracy rate. Subjects: 4 healthy volunteers. | No limitations were reported. |
