# Supplementary material for: Performance of Oral Cavity Sensors: A Systematic Review
Source: Sensors (Basel). 2023 Jan 4;23(2):588. doi: 10.3390/s23020588 (PMC9862524; doi:10.3390/s23020588)
Supplement: Supplementary file 1 [file sensors-23-00588-s001.zip › Table S3 - Summary of Physical medicine and rehabilitation Studies.pdf]

TABLE S3

SUMMARY OF PHYSICAL MEDICINE AND REHABILITATION STUDY CHARACTERISTICS SORTED BY RESEARCH TOPIC, SENSOR TYPE AND AUTHOR

Author, year	Research Topic	Application	Sensor Technology	Technical Approach	Evaluation	Limitations
de Almeida e Bueno, 2021 [106]	Athletic performance monitoring	Estimate perceived exertion	Personalized ethylene–vinyl acetate mouthguard instrumented with an electronic sensor board. The sensor board contains a microprocessor, Bluetooth, a digital microphone and an SD memory card. Board was powered by a 100mAh lithium-ion battery and was positioned in the palate.	Participants wore both the instrumented mouthguard and a metabolic system and performed an experimental protocol containing three running tests	Metric: Root mean-squared error and normalized root-mean-squared error. Subjects: 8 healthy adults.	Small sample size. Results are not generalizable to real-world experience and professional athletes.
Jiang, 2022 [104]	Speech and swallowing disorders	Rehabilitation of tongue motor control	Essix-type dental retainer with a 3x4 electrode array on a flexible PCB. Electrodes were manufactured by immersion gold plating, with protrusions with lead-free solder. Each bipolar electrode was as a pair of contacts, with 1-mm width each, 4-mm length, and 1.5-mm spacing in between.	Subjects were randomly divided into different groups and completed the visual and electro-tactile feedback training in alternating order.	Metric: Mean error rate and accuracy. Subjects: 8 healthy adults.	Small sample size. Results are not generalizable to the target population. Sensitivity to stimulation can affect results. Long-term learning, sensor placement and combined training modes were not investigated.
Caltenco 2012 [98]	Tongue-computer interface	Computer interface for paralyzed individuals.	Three versions of a copolyester palatal plate are cabled to external electronics. One with nine air-cored coils and two with 24 coils printed in five circuit board coil pads. Coils track a cylindrical stainless-steel unit (4×2mm) glued to the tongue.	Compared throughput of 3 designs of mouthpieces with tongue keyboards and pointers on typing several repetitive series designed to activate different tongue movements.	Metric: Throughput based on speed and accuracy. Subjects: 20 healthy volunteers.	The cable connection may have affected performance. Flat coil pads generate more errors due to a lack of tactile cues. Visual feedback can also improve performance.
Caltenco 2014 [99]	Tongue-computer interface	Computer interface for paralyzed individuals.	Two layouts of a copolyester palatal plate cabled to external electronics. The palatal plates had 24 coils printed in five circuit board coil pads. Coils are activated with a cylindrical stainless-steel unit (4×2mm) glued to user’s tongue.	Compared throughput and accuracy of designs of mouthpieces on typing several repetitive series and pointing using only mousepad functionality.	Metric: Percentage error. Subjects: 10 healthy volunteers.	More participants were not recruited due to the costs of making mouthpieces. Involuntary activations and temperature variations affect device performance.
Mohammadi 2019 [100]	Tongue-computer interface	Computer interface for paralyzed individuals.	A wireless, battery-driven, custom-fit copolyester palatal plate with 18 coils (10 on a keypad area and 8 on a mousepad area). Coils were printed in 10-layer circuit boards. Coils are activated with a cylindrical stainless-steel unit (4×2mm) glued to user’s tongue.	Compared performance on multi-directional tapping test of tongue interface used in the mouth and on the hand against standard gamepad joystick.	Metric: Throughput based on speed and accuracy; root-mean-square error. Subjects: 2 healthy volunteers.	Differences in the measurement method can lead to significant variations in the result.
Struijk 2017 [101]	Tongue-computer interface	Computer interface for paralyzed individuals.	A wireless, battery-driven, custom-fit copolyester palatal plate with 18 coils (10 on a keypad area and 8 on a mousepad area) printed in 10-layer circuit boards. Coils are activated with a stainless-steel unit glued or pierced to the user’s tongue.	Compared performance of keypad and mousepad typing in several sessions typing texts designed to cover most of the alphabet using tongue interface.	Metric: Percentage error. Subjects: 6 mixed volunteers.	Typing methodologies and user experience may affect performance. Production costs and participants' requirements limited the study. The pierced activation unit is challenging learning.
Park 2014 [75]	Tongue-computer interface	Computer interface for paralyzed individuals.	An acrylic arch-shaped dental retainer containing two 28 × 14 × 2mm trapezoidal circuit boards and one 5.5mm wide flat cable. The system has four 3-axis magnetometers tracking a magnetic tracer on the tongue and	Measured throughput of tongue interface based on magnetic sensors in maze navigation and centre-out tapping tasks.	Metric: Throughput based on speed and accuracy. Subjects: 2 healthy volunteers.	Not tested mechanical stress and ageing inside of the oral environment. The device needs improvements in thickness and volume, and a transmitter to

			a wireless transmitter.			compensate for variations and reduce power loss.
Amin 2020 [66]	Tongue-computer interface	Computer interface for paralyzed individuals.	A mouthguard with two 5.5 × 12.5mm white LEDs, a cylindrical endoscope camera (7 × 43mm) with 6 white LEDs and a 30° angled mirror. The image resolution is 640x480.	Performed several sessions of typing 5 sequences of characters and words using a tongue pointer with an endoscope camera.	Metric: Percentage error. Subjects: 1 healthy volunteer.	The system is wired, and obtrusive and forces users to keep their mouths open. The influence of environmental light intensity was not evaluated.
de Almeida e Bueno, 2022 [78]	Vital sign monitoring	Measure intraoral temperature	Personalized ethylene–vinyl acetate mouthguard instrumented with a custom-designed data acquisition system. The data acquisition system contained a microprocessor, Bluetooth, and a silicon-based digital temperature sensor with ±0.1 °C manufacturer accuracy from +30 to +50 °C. The board was wired to a sensor evaluation system connected to a PC.	The sensor board was placed at the buccal side of the upper first molar. The sensor was tested against a gold reference at three different temperatures in a controlled water bath. The sensors are also tested on one human volunteer.	Metric: Mean absolute error and root mean squared error. Subjects: 1 healthy adult.	Wired fabrication limited experimentation with more devices and subjects. Ambient factors were not controlled. Reference and tested sensors had resolutions of the same magnitude.
Morán-Navarro 2019 [81]	Vital sign monitoring	Measure body temperature	Commercially available electronic temperature sensor with a thermocouple calibrated to ± 0.1°C within a range of 35.5-42°C.	Compared oral, skin and tympanic temperature before, during and after exercise in the heat, controlling air-cooling and sweating against ingestible thermometer.	Metric: Bland-Altman analysis. Subjects: 12 healthy volunteers.	No limitations were reported.
