# Supplementary material for: Performance of Oral Cavity Sensors: A Systematic Review
Source: Sensors (Basel). 2023 Jan 4;23(2):588. doi: 10.3390/s23020588 (PMC9862524; doi:10.3390/s23020588)
Supplement: Supplementary file 1 [file sensors-23-00588-s001.zip › Table S4 - Summary of Internal medicine Studies.pdf]

TABLE S4

SUMMARY OF INTERNAL MEDICINE STUDY CHARACTERISTICS

Author, year	Application	Sensor Technology	Technical Approach	Evaluation	Limitations
Tiba 2021 [64]	Measure tissue blood SpO2	A Resonance Raman spectroscopy microvascular oximeter using 405 nm, 4mW laser. The sensor illuminates an area of tissue that is approximately 12mm2. The sensor is fitted with a clip inside the mouth on the buccal mucosa	Tissue haemoglobin oxygenation measurements were performed and compared with central venous oxygen saturation collected by standard clinical protocols. Clinical utility was assessed by five specialists.	Metric: Fleiss' Kappa and Modified Clarke Error Grid. Subjects: 138 patients.	No repeated measures. Comparison with central venous oxygen saturation may be oversimplified.
Smith 2004 [84]	Measure body temperature	One battery-powered and one solar-powered portable unit for continuous monitoring of temperature data. Both units contained thermistors calibrated to ± 0.1°C and were encased in polyurethane tubing.	Compared temperature measurements of different electronic temperature sensors in the mouth and axilla against pulmonary artery temperature.	Metric: Bland-Altman analysis and mean error. Subjects: 35 adult patients.	The thin, plastic, single-use disposable sheaths used can be a possible negative influence
