# Supplementary material for: Performance of Oral Cavity Sensors: A Systematic Review
Source: Sensors (Basel). 2023 Jan 4;23(2):588. doi: 10.3390/s23020588 (PMC9862524; doi:10.3390/s23020588)
Supplement: Supplementary file 1 [file sensors-23-00588-s001.zip › Table S5 - Summary of Obstetrics Studies.pdf]

TABLE S5

SUMMARY OF OBSTETRICS STUDY CHARACTERISTICS

Author, year	Application	Sensor Technology	Technical Approach	Evaluation	Limitations
Hutton 2009 [82]	Measure body temperature	Three temperature systems. Two commercially available portable digital temperature monitoring units. One thermistor based and one thermocouple based. Both digital sensors were calibrated to ± 0.2°C. One heat-sensitive, single-use, plastic strip calibrated to ± 0.1°C.	Compared measurements in different body places using an electronic and a disposable thermometer against the hospital reference thermometer	Metric: Bland-Altman analysis. Subjects: 36 mothers and 36 newborns.	The study observed predominately healthy ranges of body temperature. The study was not blinded to readings, although bias was reduced.
