# Supplementary material for: Performance of Oral Cavity Sensors: A Systematic Review
Source: Sensors (Basel). 2023 Jan 4;23(2):588. doi: 10.3390/s23020588 (PMC9862524; doi:10.3390/s23020588)
Supplement: Supplementary file 1 [file sensors-23-00588-s001.zip › Table S6 - Summary of Aerospace medicine Studies.pdf]

TABLE S6

SUMMARY OF AEROSPACE MEDICINE STUDY CHARACTERISTICS

Author, year	Application	Sensor Technology	Technical Approach	Evaluation	Limitations
Amini 2016 [62]	Measure blood SpO2	A near-infrared spectra blood oximeter probe encased in silicon. The probe consists of two light guides (1 mm diameter and separation), a 30W halogen light and a spectrometer.	Compared readings from near-infrared spectra probe against laser doppler flowmeter and reflectance pulse oximeter inside a hypobaric chamber	Metric: Root-mean-squared error. Subjects: 6 healthy volunteers	Did not compare with arterial oxygen saturation. Probe fixation was not stable, resulting in motion artefacts and saliva accumulation.
